# Multienzyme Coimmobilization on Triheterofunctional Supports

**DOI:** 10.1021/acs.biomac.2c01364

**Published:** 2023-01-17

**Authors:** Javier Santiago-Arcos, Susana Velasco-Lozano, Fernando López-Gallego

**Affiliations:** † Heterogeneous Biocatalysis Laboratory, CIC biomaGUNE, Edificio Empresarial “C”, Paseo de Miramón 182, 20009 Donostia, Spain; ‡ Instituto de Síntesis Química y Catálisis Homogénea (ISQCH-CSIC), Universidad de Zaragoza, C/ Pedro Cerbuna, 12, 50009 Zaragoza, Spain; § Aragonese Foundation for Research and Development (ARAID), 50018 Zaragoza, Spain; ∥ IKERBASQUE, Basque Foundation for Science, 48009 Bilbao, Spain

## Abstract

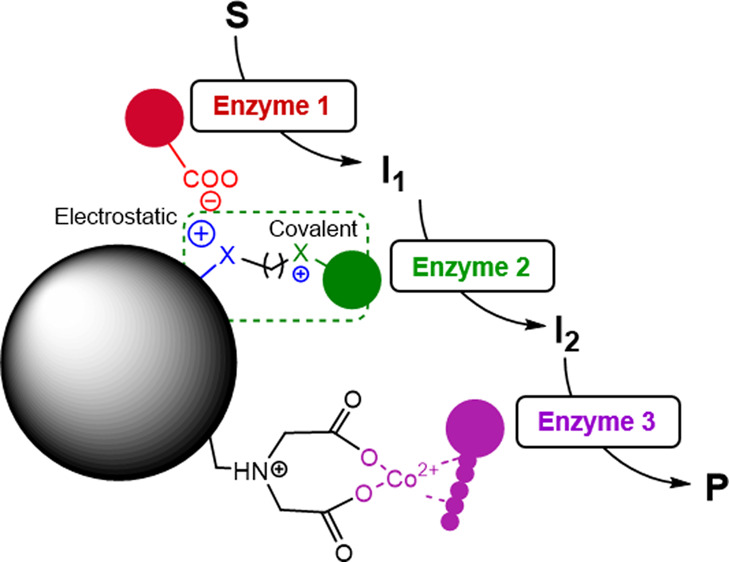

Immobilized multienzyme systems are gaining momentum in applied biocatalysis; however, the coimmobilization of several enzymes on one carrier is still challenging. In this work, we exploited a heterofunctional support activated with three different chemical functionalities to immobilize a wide variety of different enzymes. This support is based on agarose microbeads activated with aldehyde, amino, and cobalt chelate moieties that allow a fast and irreversible immobilization of enzymes, enhancing the thermostability of most of the heterogeneous biocatalysts (up to 21-fold higher than the soluble one). Furthermore, this trifunctional support serves to efficiently coimmobilize a multienzyme system composed of an alcohol dehydrogenase, a reduced nicotinamide adenine dinucleotide (NADH) oxidase, and a catalase. The confined multienzymatic system demonstrates higher performance than its free counterpart, achieving a total turnover number (TTN) of 1 × 10^5^ during five batch consecutive cycles. We envision this solid material as a platform for coimmobilizing multienzyme systems with enhanced properties to catalyze stepwise biotransformations.

## Introduction

Enzyme immobilization is a well-spread and capitalized technique exploited in a broad variety of biocatalytic industrial applications such as drug development, chemical synthesis, energy and fuel production, polymer synthesis, biomedicine and biosensors, food, and cosmetics.^1^ Confined in a defined space, immobilized enzymes remain as a heterogeneous biocatalyst in the reaction mixture, thus simplifying its separation and recycling.^2^ Beyond these advantages, enzyme immobilization has been extensively employed to enhance enzyme stability^3,4^ and to control/modulate enzyme catalytic properties.^5^ During the last 50 years, separately immobilized enzymes have been the most reported ones, whereas Systems Biocatalysis is encouraging new efforts to immobilize several enzymes on the same particle (coimmobilization) to finally create artificial biosynthetic routes.^6^

Multistep biocatalysis allows running enzyme cascades into one-pot reaction systems, which compared with multipot cascade approaches minimizes the reaction steps, reduces the byproduct formation, decreases the accumulation of toxic or unstable intermediates, shifts the thermodynamic equilibrium toward the target product, in situ recycling of enzyme cofactors if required, and ultimately increases the productivity, the titer, and the cost-efficiency of the bioprocess.^7^ However, cascade biotransformations must overcome several obstacles related to the different stabilities and reactivity requirements of each enzyme forming the system.^8^ Enzyme coimmobilization is a recurrent strategy to solve some of these drawbacks, ideally providing a suitable compartmentalized microenvironment where the enzymes may be spatially organized at the right density to increase the overall cascade efficiency, easing reaction workups, and enabling the biocatalyst reutilization.^9^ Bringing enzymes together inside solid materials may enhance the mass transport and increase the local concentration of intermediates between the coimmobilized enzymes.^10^ The benefits of enzyme coimmobilization have clearly arisen for those multienzyme systems that demands either the in situ cofactor recycling or byproduct removal.^11,12^ However, the coimmobilization of two or more enzymes on the same support is not trivial as one immobilization strategy might be beneficial for one enzyme but detrimental for the other(s). Therefore, coimmobilization by itself does not guarantee the activity and stability of a heterogeneous multienzyme system.^13^ In this context, heterofunctional supports contain several functionalities (reactive groups) on their surface that react with several surface residues (Lys, Cys, Asp, etc.) of one or more enzymes under different conditions (pH, ionic strength, temperature). These heterofunctional supports emerge as an excellent solution to coimmobilize multienzyme systems on the same surface where each enzyme is attached to the support through its optimal immobilization chemistry. The vast majority of heterofunctional supports offer the combination of only two reactive groups: one (i.e., ionic, hydrophobic, metal chelate groups) to drive the enzyme adsorption and the other (i.e., epoxy, aldehyde, glyoxyl, and vinyl groups) to react with the exposed nucleophilic residues on the enzyme surface to form covalent and irreversible bonds.^14,15^ The combination of these two groups allows a two-step enzyme immobilization, in which the enzyme is first absorbed very quickly to the support (close contact) and then irreversible covalent attachment between the enzyme and the support is formed.^16^ Although heterofunctional supports have been mainly harnessed to accomplish the multivalent covalent immobilization of single enzymes at mild conditions, recent trends are more focused on their use as a chassis for the coimmobilization of multienzyme systems controlling their spatial organization.^12,13,17^

**Scheme 1:**
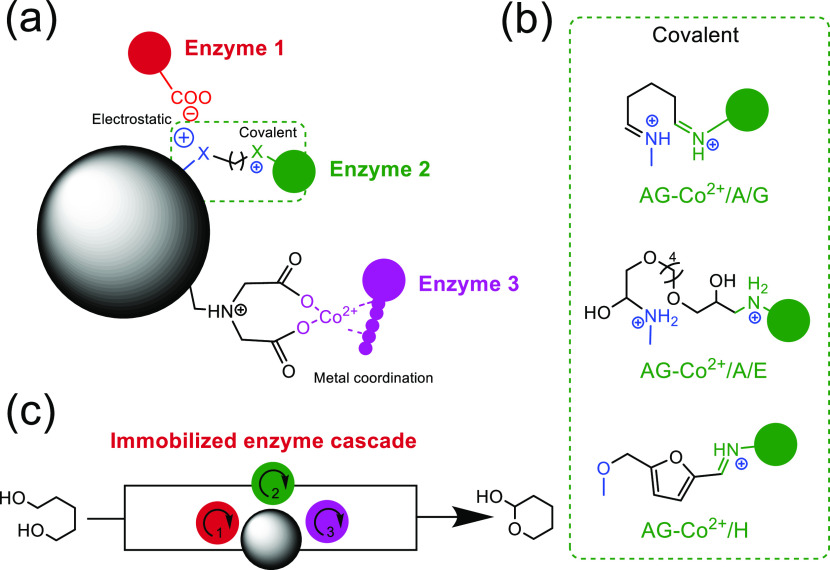


In this work, we have exploited and characterized porous supports functionalized with three reactive groups: metal chelates to site-directed immobilize His-tagged enzymes, cationic amines to ionically absorb them, and electrophile groups (aldehydes or epoxides) to promote their multivalent covalent attachment to the support surface (Scheme 1a,b). While the cationic amines and the metal chelates establish reversible bonds between the immobilized enzymes and the support surface, the electrophile ones do establish irreversible bonds that may avoid the enzyme leakage during the biocatalyst utilization. A similar trifunctional support was reported for the immobilization of a sole enzyme but never intended for the coimmobilization of a multienzyme system.^18^ We immobilized a pallet of six different enzymes on this trifunctional support and characterized their immobilization kinetics, stability, and structural rearrangements upon the immobilization process. To demonstrate the potential of this trifunctional support for the coimmobilization of multienzyme systems, we challenged it with a model system composed of three enzymes that orthogonally work to selectively oxidize 1,5-pentanediol into its corresponding lactol with in situ nicotinamide adenine dinucleotide (NAD^+^) regeneration and H_2_O_2_ removal (Scheme 1c).^19^ Finally, we evaluated the stability and reusability of the coimmobilized enzyme preparations in a batch reactor operated for consecutive and discontinuous reaction cycles.

## Experimental Methods

### Materials

The enzymes alcohol dehydrogenase (ADH) from *Bacillus stearothermophilus* (BsADH), reduced nicotinamide adenine dinucleotide (NADH) oxidase from *Thermus thermophilus* HB27 (TtNOX), catalase from *Bordetella pertussis* (BpCAT), and the lactonase from *Sulfolobus islandicus* (SiLAC) were produced as previously reported.^20^ Four percent cross-linked agarose (AG) beads (particle size 50–150 μm; pore diameter 300 nm) were purchased from Agarose Bead Technologies (Madrid, Spain); epoxy methacrylate microbeads ECR8204F (Pu) (particle size 150–300 μm; pore diameter 300–600 Å) so were kindly donated by Purolite; and Cellulose MT200 (particle size 100–250 μm; pore diameter 32 nm) was purchased from IONTOSORB (Usti and Labem, Czech Republic). Compounds such as ethylenediamine (EDA), imidazole, iminodiacetic acid, cobalt chloride, sodium periodate, sodium hydroxide fluorescein isothiocyanate (FITC), rhodamine B isothiocyanate, sodium acetate, sodium chloride, sodium phosphate, sodium bicarbonate, glutaraldehyde (GA), SYPRO Orange Protein Gel Stain, 1,5-pentanediol, 5-hydroxypentanal, tetrahydro-2*H*-pyran-2-ol, δ-valerolactone, as well as the enzymes catalase from bovine liver (BlCAT) and alcohol dehydrogenase from horse liver (HlADH) were acquired from Sigma-Aldrich Chemical Co. (St. Louis, IL). All other reagents were of analytical grade.

### Preparation of Triheterofunctional Support Activated with Cobalt Chelates, Secondary Amine Groups, and Aldehydes (AG-Co^2^/A/G)

For the first step, we prepared epoxy-activated agarose (AG-E) as described elsewere.^15^ Then, we activated the AG-E with iminodiacetic acid (AG-E/IDA) by preparing a suspension of 10 g (≈14 mL) of AG-E in 100 mL of 0.5 M iminodiacetic acid at pH 11. The suspension was maintained under gentle agitation at 200 rpm for 1 h at room temperature (RT). Afterward, the support was filtered and rinsed with 10 volumes of water. Once AG-E/IDA was obtained, we introduced amino groups by incubating it overnight with 10 volumes of 2 M ethylenediamine at pH 11 (AG-A/IDA) under gentle agitation at 200 rpm at room temperature. Later, the support was filtered and gently rinsed with water. Then, the introduction of aldehyde moieties was conducted by incubating the support overnight with a 15% glutaraldehyde solution in a 200 mM sodium phosphate buffer pH 7 (AG-IDA/A/G) under gentle agitation at 200 rpm at room temperature. Once the incubation concluded, the support was filtered and washed with at least 10 volumes of water. Finally, to introduce the metal group, the support was incubated with 10 volumes of 30 mg·mL^–1^ of CoCl_2_ for 2 h at room temperature (AG functionalized with GA, EDA, and IDA/cobalt groups (AG-Co^2+^/A/G)). In the end, the support was filtered and washed with abundant water and stored at 4 °C protected from light (Scheme S1).

### Preparation of Triheterofunctional Support Activated with Cobalt Chelates, Secondary Amine Groups, and Epoxides (AG-Co^2+^/A/E)

One volume of AG-A/IDA was suspended in 20 volumes of 0.15 M of 1,4-butanediol diglycidyl ether (BD), 12% acetone in 80 mM bicarbonate buffer pH 9 and incubated overnight under gentle agitation at 200 rpm at room temperature. Once the incubation concluded, the support was filtered and washed with at least 10 volumes of 20% acetone in water and then with only water. Finally, to introduce the metal group, the support was incubated with 10 volumes of 30 mg·mL^–1^ of CoCl_2_ for 2 h at room temperature (AG-Co^2+^/A/E). In the end, the support was filtered and washed with abundant water and stored at 4 °C protected from light (Scheme S2). Homogeneous suspensions of AG-Co^2+^/A/E microbeads were obtained by applying short, repeated water bath ultrasound vibrations (10 s) to homogenize the formed microbead suspension until no visible aggregates were observed.

### Preparation of Biheterofunctional Support Activated with Cobalt Chelates and Hydroxymethylfurfural (HMF) AG-Co^2+^/H

One volume of AG-E/IDA was suspended in 10 volumes of 100 mM of hydroxymethylfurfural (HMF) in 100 mM sodium phosphate buffer pH 8 and incubated overnight under gentle agitation at 200 rpm at RT (AG-H/IDA). Once the incubation concluded, the support was filtered and washed with at least 10 volumes of water. Finally, to introduce the metal–ligand, the support was incubated with 10 volumes of 30 mg·mL^–1^ of CoCl_2_ for 2 h at room temperature (AG-Co^2+^/H). In the end, the support was filtered and washed with abundant water and stored at 4 °C protected from light (Scheme S3).

### Preparation of Triheterofunctional Supports CEL-Co^2+^/A/G and Pu-Co^2+^/A/G

The functionalization of cellulose microbeads (CEL) and polymethacrylate microbeads (Pu) was done following the same activation protocol to prepare AG-Co^2+^/A/G but replacing AG with CEL (CEL-Co^2+^/A/G) or Pu microbeads (Pu-Co^2+^/A/G). While the Pu microbeads are commercially supplied with epoxides (ECR8204F), cellulose was activated with the epoxy groups following the same protocol as for agarose.^15^

### Degree of Activation of the Supports

#### Epoxy Group Quantitation

Epoxy groups were quantified indirectly through the oxidation of the diols resulting from epoxide ring opening under acid conditions. These diols were then titrated with NaIO_4_ as described elsewhere.^21^ Briefly, 1 g of the support was incubated with 10 mL of 0.5 M H_2_SO_4_ for 1 h at room temperature to hydrolyze the epoxy groups. Afterward, the hydrolyzed support (yielding vicinal diols) was oxidized with 10 mM of NaIO_4_ (1:10 suspension) by incubation at room temperature (typically 1–2 h). The number of epoxy groups was calculated by the difference in the NaIO_4_ consumption between the hydrolyzed support and the initial epoxy support. Different consumption degrees of periodate were quantified by titration with potassium iodide (KI). Briefly, 20 μL of remnant NaIO_4_ in the supernatant was mixed with 200 μL of 10% KI in saturated bicarbonate solution and measuring the absorbance at 405 nm.

#### IDA Group Quantitation

IDA groups were indirectly quantified following the same hydrolysis procedure as for epoxy group quantitation but hydrolyzing both AG functionalized with epoxide and IDA groups (AG-IDA/E) and AG-E with H_2_SO_4_ for 1 h at room temperature to hydrolyze the epoxy groups. Once epoxy groups were hydrolyzed, the formed diols were measured by oxidative titration with NaIO_4_. IDA groups were calculated as the difference in the NaIO_4_ consumption of the hydrolyzed both AG-E (before IDA group introduction) and AG-IDA/E. The consumption of periodate was spectrophotometrically measured by titration with KI as mentioned above.

#### Amino Group Quantitation

Introduced amino groups were quantified by titration with picrylsulfonic acid.^22^ Briefly, 0.25 mL of picrylsulfonic acid solution (5% w/v) diluted 500 times in 100 mM sodium bicarbonate buffer pH 8.5 (Cat. P2297, Sigma-Aldrich) was mixed with 0.5 mL of a 1:10 suspension of AG-IDA/A in 100 mM sodium bicarbonate buffer pH 8.5. The mixture was incubated at 37 °C for 2 h with gentle mixing. Afterward, the support was washed 4 times with 0.75 mL of 1 M NaCl followed by five washes of 0.75 mL of distilled water. The washed support was resuspended 1:10 in distilled water to measure the absorbance of 200 μL at 335 nm against a support control without amino functionalization (AG-IDA/E). The calibration curve of ethylenediamine (linear range 0.03–0.25 mM) was also prepared under the same conditions. Micrographs of the picrylsulfonic-activated AG-IDA/E were acquired by placing 100 μL of a 1:200 water suspension of the support in water in a 96-well clear-bottom black microplate and visualized using a Plan Fluorite 4× phase objective and performed color brightfield imaging, multicolored light-emitting diode (LED) illumination within a Cytation5 Cell Imaging Reader (BioTek Instruments) (Figure S1A).

#### Aldehyde Group Quantitation

Aldehyde functionalization with glutaraldehyde was quantified by titration with Schiff’s reagent.^23^ Briefly, 20 μL of Schiff’s reagent (Cat. 1.09034, Sigma-Aldrich) was mixed with 200 μL of a 1:10 suspension of AG-Co^2+^/A/G in distilled water. The mixture was incubated at room temperature for 30 min with gentle mixing. Later, the support was washed four times with 0.5 mL of 1 M NaCl followed by five washes of 0.5 mL of distilled water. The washed support was resuspended 1:10 in distilled water to measure the absorbance of 100 μL at 570 nm against a support control without GA activation (AG-IDA/A). Calibration curve of glutaraldehyde (linear range 0.15–2.5 mM) was also prepared under the same conditions. Micrographs of the Schiff-activated AG-IDA/A were acquired by placing 100 μL of a 1:200 water suspension of the support in water in a 96-well clear-bottom black microplate and visualized using a Plan Fluorite 4× phase objective and performed color brightfield imaging, multicolored LED illumination within a Cytation5 Cell Imaging Reader (BioTek Instruments) (Figure S1B).

#### HMF Group Quantitation

Agarose functionalization with HMF was spectrophotometrically quantified by using a HMF calibration curve (linear range 0.19–1.56 mM). To properly determine the calibration curve, we measured the absorbance of 200 μL of a suspension (1:20) of AG-IDA/H or AG incubated with different concentrations of HMF in water at 285 nm in a Microplate Reader Epoch 2 (BioTek Instruments).

#### Enzyme Immobilization

The immobilization was conducted by mixing 10 mL of enzyme solution (in 100 mM sodium phosphate buffer pH 7) with 1 g of support (AG-Co^2+^/A/G or AG-Co^2+^/A/E or AG-Co^2+^/H). The suspension was maintained under gentle agitation at 25 rpm at 4 °C. The immobilization course was followed by measuring the activity for both the supernatant and the suspension. Once the immobilization was completed (typically 30 min), the immobilization mixture was incubated for 2 h in total (including the immobilization time) at 25 rpm and 4 °C to promote the formation of multivalent attachment between the nucleophiles on the enzyme surface (mainly Lys) with either the aldehydes or the epoxide of the support surface. Subsequently, a blocking step was done by the addition of glycine (1 M, pH 8) followed by soft agitation overnight at 25 rpm and 4 °C. Once the support was blocked, the immobilized sample was washed five times with five volumes of 25 mM sodium phosphate buffer pH 8, filtered, and stored at 4 °C.

#### Enzyme Coimmobilization

Enzyme coimmobilization was conducted following the same methodology previously described but incorporating the enzymes in two different orders. For the sequential immobilization (heterogeneous biocatalysts number one, HB1), 10 mL of TtNOX in 100 mM sodium phosphate buffer at pH 7 was first incubated with 1 g of AG-Co^2+^/A/G for 2 h at 25 rpm and 4 °C. Afterward, the suspension was filtered and 10 mL of a solution of BlCAT in the same buffer was added, followed by incubation for 2 h at 4 °C and 25 rpm. Later, the suspension was filtered again and 10 mL of BsADH in the same buffer was added and incubated for 2 more hours at 4 °C and 25 rpm. Then, the suspension was filtered and incubated overnight at 4 °C and 25 rpm with 1 M glycine at pH 8 to block the remaining aldehyde groups. Finally, the biocatalyst was filtered and washed with 25 mM sodium phosphate buffer pH 7 and stored at 4 °C. For the coimmobilization incorporating the three enzymes at the same time (heterogeneous biocatalysts, HB2, HB2-AG, HB2-Pu, and HB2-CE), the immobilization was conducted as mentioned above but incorporating the three enzymes at the beginning (2 h of immobilization time).

#### Enzyme Activity Assays

Enzyme activities were spectrophotometrically measured in transparent 96-well microplates with a flat bottom (Nunc), employing a Microplate Reader Epoch 2 (BioTek Instruments) provided with the software Gen5.

#### ADH Activity

Two hundred microliters of a reaction mixture containing 10 mM of 1,5-pentanediol and 1 mM of NAD^+^ in sodium phosphate buffer at pH 8 were incubated with 5 μL of enzymatic solution or 10 μL of suspension (properly diluted) at 30 °C. The increase in the absorbance at 340 nm due to the reduction of NAD^+^ was recorded. One unit of activity was defined as the amount of enzyme that was required to reduce 1 μmol of NAD^+^ to NADH per minute at the assayed conditions.

#### NADH Oxidase Activity

Two hundred microliters of a reaction mixture containing 0.2 mM of NADH and 150 μM of flavin adenine dinucleotide (FAD^+^) in 50 mM sodium phosphate buffer pH 8 at 30 °C were incubated with 5 μL of enzymatic solution or 10 μL of suspension (properly diluted) at 30 °C. The oxidation of NADH was monitored as a decrease in the absorbance at 340 nm. One unit of activity was defined as the amount of enzyme that was required to oxidize 1 μmol of NADH to NAD^+^ per minute at the assayed conditions.

#### Catalase Activity

Two hundred microliters of a reaction mixture containing 35 mM of hydrogen peroxide in 100 mM sodium phosphate pH 8 at 30 °C were incubated with 5 μL of the enzymatic solution or 10 μL of suspension (adequately diluted). The catalase activity was measured by recording the decrease in the absorbance at 240 nm. One unit of CAT activity was defined as the amount of enzyme required for the disproportionation of 1 μmol of hydrogen peroxide per minute at the assessed conditions.

#### Lactonase Activity

Lactonase activity was indirectly monitored by the decrease in the pH triggered by the formation of 5-hydroxypentanoic acid from its corresponding lactone hydrolysis. Briefly, 200 μL of a reaction mixture containing 1 mM δ-valerolactone, 0.1% acetonitrile, and 0.25 mM *p*-nitrophenol in 2.5 mM sodium phosphate buffer at pH 7.0 was incubated with 5 μL of enzymatic solution or 10 μL of suspension (properly diluted) at 30 °C. The decrease in the absorbance of *p*-nitrophenol (pH indicator) at 410 nm was recorded. One unit of activity was defined as the amount of enzyme that was required to produce 1 μmol of 5-hydroxypentanoic acid (titrated by pH change) per minute at the assayed conditions.

#### Thermal Inactivation

Thermal inactivation kinetics of the biocatalysts were conducted by incubating a solution or a suspension of the free or immobilized enzymes in 100 mM sodium phosphate buffer pH 8.0 at the indicated temperature until more than 50% of the initial activity was lost. To calculate half-life times, the obtained experimental measurements were adjusted to a three-parameter biexponential kinetic inactivation model.^24^ Additionally, we determined the thermal denaturation temperature (*T*_m_) of the biocatalysts by fluorescent thermal shift assay. Briefly, 25 μL of 1 μM enzyme solution or suspension in 25 mM sodium phosphate buffer pH 8 containing 5 μL 60× of SPYRO Orange Protein Gel Stain was placed into a 200 μL clear thin-wall polypropylene eight-tube strip for polymerase chain reaction (PCR). The protocol was set with a temperature analysis range from 25 to 95 °C in 1 h, recording the fluorescence in a CFX Real-Time PCR system (Bio-Rad). Raw fluorescence data were analyzed to determine the denaturation temperature (*T*_m_) from nonlinear fitting of thermal denaturation data^25^ employing OriginLab software.

#### Protein Labeling with Fluorescent Probes

Fluorescent labeling was done accordingly with a methodology reported elsewhere.^26^ An enzyme solution (typically 0.25 mg·mL^–1^) in 100 mM of sodium bicarbonate buffer at pH 8.5 was mixed (1:10 molar ratio) with either rhodamine B isothiocyanate or fluorescein isothiocyanate (FITC) in dimethyl sulfoxide (DMSO) (5 mg·mL^–1^) and incubated 1 h with gentle agitation at 25 °C in darkness. Afterward, the remaining fluorophore was eliminated by dialysis through a centrifugal filter unit (cutoff of 10 kDa) with 25 mM sodium phosphate buffer pH 8.0.

#### Confocal Laser Scanning Microscopy (CLSM) Imaging

The distribution of immobilized fluorophore-labeled proteins was analyzed with a confocal microscope Espectral ZEISS LSM 510 with an excitation laser (λ_ex_: 561 nm) and emission filter (LP575). Confocal imaging was carried out at both 20× and 40× (water, 1.2 NA) objectives and a 1:200 (w/v) buffered suspension in 25 mM phosphate at pH 7. The resulting micrographs were analyzed with FIJI^27^ using an image analytical routine previously reported.^28^ From confocal images, we obtained an average and normalized fluorescence radius profile, using FIJI software and its plugin module for radial profile generation (developed by Paul Baggethun). Subsequently, a Gaussian fit was applied to the obtained profiles of at least 10 single beads. Subsequently, we searched for the fitted data point that corresponds to 50% of the maximum normalized fluorescence fitted peak (*y*FWHM), and the corresponding radius coordinate (*x*FWHM) to that data point was then subtracted from the radius (*R*) of the analyzed bead to finally obtain the full width at half-maximum (FWHM), which means the infiltration distance of the enzyme into the bead surface. Dividing this infiltration distance between the radius size, we obtained the relative infiltration distance.

#### Fluorescent Anisotropy

The polarized fluorescence of immobilized samples loaded with 0.5 mg of FTIC-labeled enzymes was measured to determine the fluorescence anisotropy of FTIC conjugated to the free and immobilized proteins. To calculate the anisotropy values, 3.5 ng of either free or immobilized enzymes was placed into a 96-well dark plate and measured in a Microplate Reader Synergy H1, BioTek. Anisotropy values were obtained following the methodology described elsewhere.^19^

The anisotropy values of all immobilized samples were normalized to the anisotropy of the free enzyme. Values higher than one mean enzymes with higher rotational tumbling than the free enzyme, while values lower than one mean enzymes with lower rotational tumbling than the free enzyme.

#### Intrinsic Fluorescence of Tryptophans

Immobilized biocatalysts loading 0.5 mg of protein·g support^–1^ were used for this experiment. The intrinsic fluorescence of free and immobilized His-BsADH was measured before and after the samples were incubated at 80 °C for 1 h. To that aim, 70 μg of either free or immobilized enzymes was placed in a 96-well dark plate and the fluorescence emission spectra were recorded between 300 and 500 nm upon the sample excitation at 280 nm, using emission bandwidths of 5 nm. All spectroscopic measurements were performed in 25 mM phosphate buffer at pH 7.

#### Batch Reactions and Recycling of Coimmobilized Enzymes

Heterogeneous biocatalysts (50 mg) were placed inside a capped plastic tube (2 mL) containing 300 μL of a reaction mixture consisting of 20 mM of 1,5-pentanediol, 1 mM of NAD^+^, and 0.15 mM of FAD^+^ in 100 mM sodium phosphate buffer pH 8 allowing atmospheric oxygen supplementation by punching the tap with an open needle. Reactions were incubated at 30 °C at 250 rpm inside an orbital incubator. The reaction course was monitored by withdrawing samples at periodic intervals, which were analyzed by chromatographic methods.

### Chromatographic Methods

#### Gas Chromatography (GC)

Prior to GC analysis, 50 μL of the reaction sample was mixed with 200 μL of ethyl acetate to perform a liquid–liquid extraction of the compounds of interest containing 2 mM eicosane as the external standard. After the extraction, 30–50 mg of anhydrous MgSO_4_ was added to dry samples before GC analysis. Gas chromatography analyses were carried out in an Agilent 8890 GC system chromatograph using a J&W HP-5 GC column (30 m × 0.32 mm × 0.25 μm), helium as the support gas, and equipped with a flame ionization detector (FID). The injector was set at 280 °C and the FID at 300 °C. Separation of extracted compounds in ethyl acetate was done by the following temperature program: the initial temperature at 60 °C, maintained 2 min, ramp to 160 °C at a rate of 10 °C·min^–1^, ramp 2–240 °C at a rate of 20 °C·min^–1^ and finally maintained 4 min. Retention times for 1,5-pentanediol, 5.6 min, tetrahydro-2*H*-pyran-2-ol, 3.4 min, δ-valerolactone, 5.8 min, and eicosane (external standard), 16.4 min.

#### High-Performance Liquid Chromatography (HPLC) analysis

5-Hydroxypentanal was quantified by HPLC through derivatization into its corresponding *O*-benzylhydroxylamine derivative.^29^ Briefly, 10 μL of aqueous reaction sample (0.6–20 mM) was mixed with 50 μL of *O*-benzylhydroxylamine hydrochloride (130 mM in pyridine/methanol/water 33:15:2) and incubated for 5 min at 25 °C. Afterward, 500 μL of methanol was added and then centrifuged 5 min at 13 450*g*. HPLC analysis was conducted in an Agilent Technologies 1260 Infinity II chromatograph equipped with a Poroshell EC-C18 column (4.6 × 100 mm^2^, 2.7 μm). Samples were detected at 215 nm and were eluted at 1 mL·min^–1^ flow rate employing two mobile phases: phase A composed of trifluoroacetic acid 0.1% in water and phase B composed of trifluoroacetic acid 0.095% in 4:1 acetonitrile/water. Elution conditions: 10–100% of B over 30 min, followed by 10 min to recover the initial conditions. The retention time of O-benzylhydroxylamine derivatized 5-hydroxypentanal was 14.4 min.

## Results and Discussion

### Support Functionalization

Ideally, a heterofunctional support should enable the efficient coimmobilization of different enzymes through chemistries that improve the overall properties of the multienzyme system. Inspired by a previously described triheterofunctional support exploited for the immobilization of single enzymes,^18^ we also functionalized porous agarose microbeads with cobalt chelates to site-directed immobilize His-tagged enzymes, with positively charged secondary amines to ionically absorb negatively charged enzymes and electrophile groups (aldehyde and epoxide) to react with the nucleophile residues at the enzyme surface. These three reactive groups should allow the immobilization of several different enzymes through three different chemistries (Scheme 1a) comprised of a two-step enzyme immobilization process: a first and fast enzyme binding (either through electrostatic interactions or metal-based affinity) followed by a covalent enzyme binding.

As support, we selected 4BCL porous agarose microbeads (300 nm average pore size and 90–150 μm bead diameter), which have a suitable pore diameter for the immobilization of enzymes, a suitable particle size for the use of the heterogeneous biocatalysts in both batch and flow reactors, and a great versatility to be functionalized with a plethora of reactive groups.^4^ We first functionalized agarose microbeads with epoxy groups (AG-E),^30^ and then AG-E was incubated with iminodiacetic acid (IDA) to generate the bifunctional support containing epoxy and IDA groups (AG-IDA/E) (Scheme S1). The degree of IDA functionalization is easily controlled by the pH and the incubation time (Table S1). After this step, we introduced a 1:1 molar ratio of epoxy/IDA groups on the modified agarose surface (19 and 20 μmol·g^–1^, respectively) (Table S2). Afterward, we introduced the first target functionality by incubating the AG-IDA/E with ethylenediamine (EDA), which converted the remaining epoxy groups into amine ones but maintaining intact the IDA ligands (AG-IDA/A). Then, the second functionality was introduced by incubating the AG-IDA/A with glutaraldehyde (G), a bifunctional agent, which reacts quantitatively with the primary amine of EDA, giving rise to a functionalized support with aldehyde and secondary amine groups (AG-IDA/A/G) (Scheme S1). The functionalization of AG-IDA/A with primary amines and AG-IDA/A/G with aldehydes was confirmed by titration with picrylsulfonic acid and with the Schiff reagent, respectively (Figure S1). Optical microscopy images reveal that the functionalization of the agarose microbeads is mostly uniform throughout their porous structure. Finally, we incubated AG-IDA/A/G with a cobalt chloride solution to form cobalt chelates, which are the third functional group of the trifunctional support (AG-Co^2+^/A/G) (Scheme S1). We found out that the functional groups are uniformly distributed over the porous surface of the agarose beads, enabling the enzymes to be potentially immobilized on any region (outer and inner) of the support particles. Apart from the aliphatic aldehydes, we also explored two other possible activation chemistries that functionalize the support with epoxy and aryl aldehydes (Scheme 1b). To that aim, we employed 1,4-butanediol diglycidyl ether to replace the aldehyde groups of AG-Co^2+^/A/G by epoxide ones, yielding a heterofunctional support functionalized with cobalt, amino, and epoxide groups (AG-Co^2+^/A/E; Scheme S2). On the other hand, we replaced GA and EDA with hydroxymethylfurfural, finally yielding a heterofunctional support functionalized with cobalt and aromatic aldehyde groups (AG-Co^2+^/H; Scheme S3 and Table S2). After the titration of epoxy, aldehydes, amine, and cobalt chelates, the three supports were functionalized with different reactive groups in almost equimolar ratios per gram of support and similar reactive group density but HMF, where its density was three times lower than the aldehydes and epoxy groups (Table S2). Detailed abbreviations of all of the prepared supports are provided in Table S3.

### Enzyme Immobilization on Heterofunctional Supports

We selected an enzyme panel to evaluate the immobilization efficiency of the three heterofunctional supports and the stabilization effects they promote on the immobilized enzymes. Herein, the enzyme panel studied is composed of a homotetrameric His-tagged alcohol dehydrogenase from *B. stearothermophilus* (BsADH),^31^ a dimeric alcohol dehydrogenase from horse liver (HlADH),^32^ an untagged homodimeric NADH oxidase from *T. thermophilus* HB27,^33,34^ a His-tagged homodimeric lactonase from *S. islandicus* (SiLAC),^35^ an untagged tetrameric catalase from bovine liver (BlCAT),^36^ and a His-tagged tetrameric catalase from *B. pertussis* (BpCAT), which expresses both higher specific activity and stability than BlCAT.^37^

All enzymes were immobilized on the three heterofunctional supports in less than 2 h (Figure S2). Regardless of the nature of the reactive groups displayed in the supports, His-tagged enzymes (as BsADH, BpCAT, and SiLAC) were quantitatively immobilized, in contrast to the untagged ones (BlCAT, HlADH, and NOX), which achieved slightly lower immobilization yields (>92%) (Table 1). With these results in hand, we demonstrate the feasibility of these heterofunctional supports to effectively immobilize a wide variety of His-tagged and untagged enzymes of different sizes and electrostatic surfaces under neutral pH conditions. These heterofunctional supports attain higher immobilization performance compared with agarose microbeads activated with cobalt chelates and epoxy groups (AG-Co^2+^/E), a benchmarked heterofunctional support widely used in applied biocatalysis^38^ (Table S4).

**Table 1:** Single-Enzyme Immobilization Parameters on Different Heterofunctional Activated Agarose Microbeads

enzyme	immobilization support	enzyme load (mg·g^–1^)	Ψ (%)^a^	recovered activity (U·g^–1^)/(%)^b^	half-life time (*t*_1/2_) (h)^c^
BsADH	AG-Co^2+^/A/G	0.47	100	0.42 (21)	0.51
	AG-Co^2+^/A/E	0.47	100	0.58 (30)	0.85
	AG-Co^2+^/H	0.47	100	0.37 (19)	0.42
HlADH	AG-Co^2+^/A/G	1.52^d^	99	0.24 (45)	54
	AG-Co^2+^/A/E	1.54^d^	100	0.25 (47)	21
	AG-Co^2+^/H	1.51^d^	98	0.22 (40)	26
TtNOX	AG-Co^2+^/A/G	1.23	92	0.76 (8.1)	9.2
	AG-Co^2+^/A/E	1.14	94	0.14 (1.3)	15.7
	AG-Co^2+^/H	1.16	99	0.11 (1.1)	24.0
BlCAT	AG-Co^2+^/A/G	0.54^d^	96	12 (6.5)	4.4
	AG-Co^2+^/A/E	0.52^d^	92	8 (4.3)	4.0
	AG-Co^2+^/H	0.55^d^	98	11 (6.0)	3.4
BpCAT	AG-Co^2+^/A/G	0.25	99	19 (22)	17.0
	AG-Co^2+^/A/E	0.25	100	0 (0)	25.0
	AG-Co^2+^/H	0.25	100	0.6 (0.7)	17.0
SiLAC	AG-Co^2+^/A/G	0.43	100	0.18 (42)	7.4
	AG-Co^2+^/A/E	0.43	100	0.05 (12)	4.7
	AG-Co^2+^/H	0.43	100	0.09 (21)	6.4

a Immobilization yield, Ψ = (immobilized activity/offered activity) × 100.

b % Recovered activity is defined as the coefficient between the specific activity of the immobilized enzyme and the specific activity of the soluble one × 100.

c Half-life time studies were assayed at different temperatures accordingly with each enzyme; thus, 65 °C for BsADH, 45 °C for HLAHD, 80 °C for TtNOX, 40 °C for BpCAT, 45 °C for BlCAT, and 50 °C for SiLAC. Half-life times of the free enzymes are provided in Table S5.

d Total protein content of semipure enzyme solutions. BsADH, BpCAT, and SiLAC, are His-tagged at their N-terminus, while HlADH BlCAT, and TtNOX are untagged.

For example, the positive amine groups displayed in the AG-Co^2+^/A/G and AG-Co^2+/^A/E surfaces seem to increase the immobilization yield up to >90% for enzymes with few exposed lysine residues, as is the case of TtNOX (four exposed lysine residues, PDB 1NOX), which only reached a 54% immobilization yield on AG-Co^2+^/E. On the other hand, we evaluated the performance of the different heterofunctional supports by comparing the recovered activity of each immobilized preparation. An ideal support would have a 100% recovered activity (or immobilization effectiveness of 1), while lower values indicate enzyme inactivation upon immobilization. Immobilized enzymes follow a trend of activity reduction in all cases (Table 1). Particularly, ADHs suffer a 50–80% enzyme inactivation upon their quantitative immobilization on these matrices. These results are in agreement with our previous report, where BsADH was immobilized on different epoxy-activated matrices.^19^ Unlike ADHs, the oxygen-dependent NADH oxidase (TtNOX) undergoes a marked enzyme activity reduction upon immobilization (losing more than 90% of its initial activity), recovering the highest activity when using AG-Co^2+^/A/G (8.1%). This large activity reduction effect is mainly attributed to a hampered oxygen diffusion inside the macroporous agarose structure previously reported by our group.^39^ Likewise, both catalases show a marked activity reduction upon immobilization, where BlCAT retains less than 7% of its initial activity, while BpCAT expresses a 3-fold higher residual activity only when immobilized on AG-Co^2+^/A/G (22%). This high activity loss has also been reported by other authors when immobilizing BlCAT on highly glutaraldehyde-activated agarose microbeads.^40^ Finally, the studied lactonase maintains 12–42% of its initial activity upon immobilization, where AG-Co^2+^/A/G provides the highest recovered activity of this enzyme. Previously, we coimmobilized both the SiLAC and BsADH on the same AG-Co^2+^ microbeads, recovering 100% of its initial activity upon coimmobilization by metal–ligand affinity;^41^ thus, we suggest that the enzyme inactivation of lactonase is triggered by the multivalent covalent attachment promoted by the GA groups.

Additionally, we assessed the thermal stability of the different heterogeneous biocatalysts at specific temperature conditions depending on the *T*_m_ values of the soluble enzymes (Figure S3). For example, the mesophilic BlCAT was inactivated at 45 °C because the *T*_m_ value of the free enzyme was 50 °C; however, the thermophile TtNOX was challenged at 80 °C because its *T*_m_ was 78 °C (Table S5). The immobilization stabilizes the majority of the herein tested enzymes, reaching up to 20-fold higher half-life times for some of them (i.e., the HlADH immobilized on AG-Co^2+^/A/G) (Table 1). Interestingly, TtNOX immobilized on the three different carriers underwent hyperactivation during the early times of the thermal inactivation (Figure S3A). This result may be related to structural rearrangements induced by the temperature in the quaternary structure of this thermophile enzyme.^34^ Surprisingly, immobilized BsADH, regardless of the support, is less stable than its free counterpart. The physical and chemical congruences of BsADH and support surfaces might cause protein structural distortions that lead to a less stable biocatalyst when it is supported. This specific issue we find for BsADH may be addressed by postimmobilization polymeric coatings that stabilize the quaternary structure of oligomeric enzymes.^40^ Despite the three supports efficiently immobilizing all of the tested enzymes with high yields, AG-Co^2+^/A/G proves to be the optimal one to maximize the recovered activity and stability (high *t*_1/2_ values) of a wider range of enzymes. For this reason, we select this support for further characterization studies.

### Orthogonality of the Immobilization Chemistries in the Trifunctional Support AG-Co^2+^/A/G

AG-Co^2+^/A/G displays three different reactive groups that may immobilize enzymes through three different mechanisms to prepare immobilized multienzyme systems with industrial relevancy. Herein, we select three different enzymes whose immobilization requires three different chemistries; His-BsADH, TtNOX, and BlCAT. In particular, TtNOX hardly interacts with cationic exchangers,^34^ unlike BlCAT, which strongly does with positively charged supports.^42^ On the other hand, metal chelates bind His-BsADH very efficiently and TtNOX has been successfully immobilized on supports functionalized with aldehyde as a standalone reactive group.^34^ Understanding the mechanism that drives the immobilization of each enzyme will allow us to design more efficient coimmobilization protocols to fabricate highly active and stable multifunctional heterogeneous biocatalysts. The combination of these three enzymes presents a great potential in applied biocatalysis not only for the selective oxidation of diols into their corresponding aldehydes or lactols,^19^ lactones, and ω-hydroxy acids^41^ but also for the synthesis of amino alcohols when coupled to transaminases.^20^

**Figure 1:**
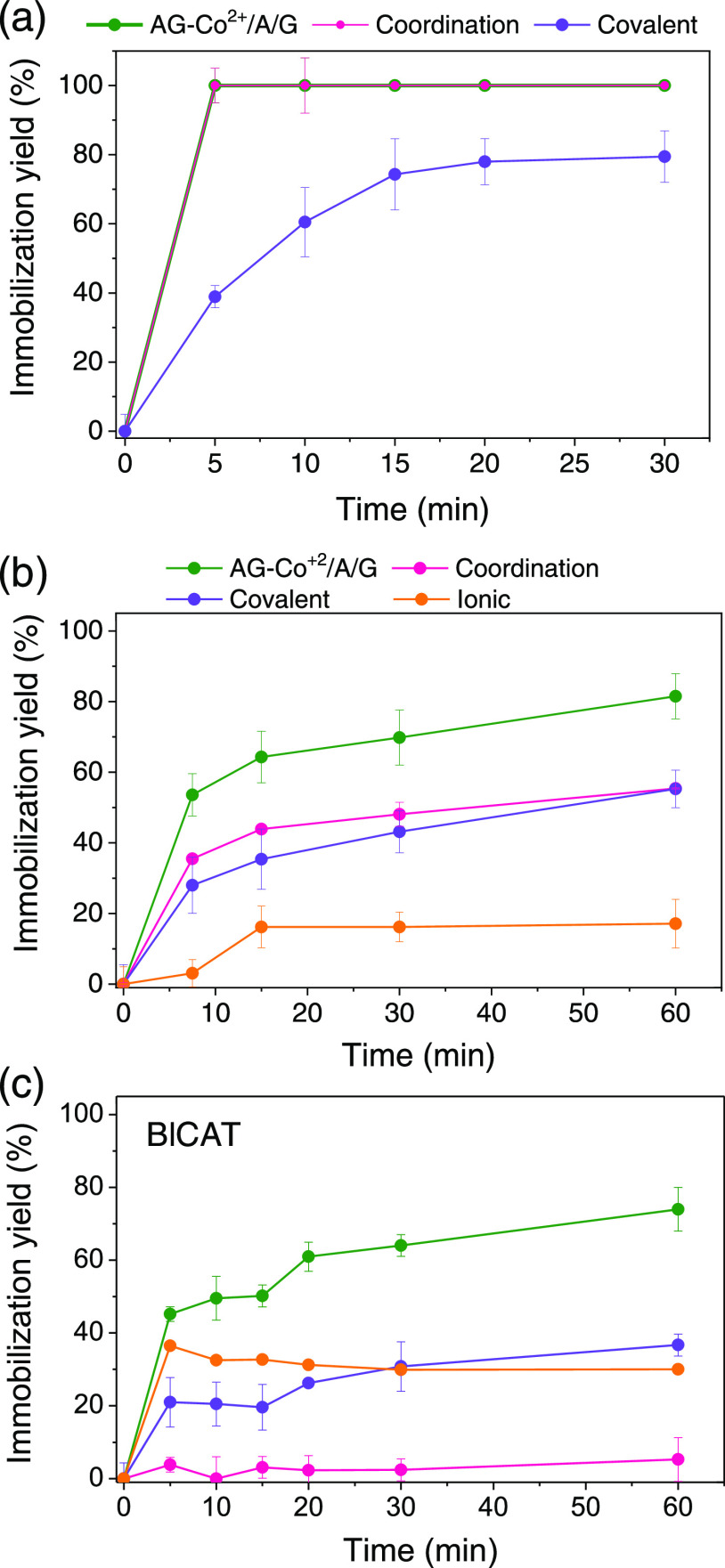
Driving immobilization chemistry of different enzymes, (a) histidine-tagged BsADH, (b) untagged TtNOX, and (c) untagged BlCAT, on triheterofunctional activated agarose microbeads. For AG-Co^2+^/A/G, the immobilization was carried out at 100 mM sodium phosphate buffer pH 8 (green lines and symbols). To only assess the coordination chemistry (pink line), the immobilization was conducted by previously blocking the G groups of AG-Co^2+^/A/G (with glycine 1 M for 16 h) and performing the immobilization at 1 M NaCl. To only assess the covalent chemistry (purple line), the immobilization was conducted at 0.3 M imidazole and 1 M NaCl. To only assess the ionic chemistry (orange line), the immobilization was conducted by previously blocking G groups of AG-Co^2+^/A/G (with glycine 1 M for 16 h) and performing the immobilization at 0.3 M imidazole. The green line represents the immobilization course on AG-Co^2+^/A/G where the three chemistries can participate. In all cases, the immobilization was conducted at 4 °C and 25 rpm.

To that aim, we evaluated the individual contribution of each reactive group displayed in AG-Co^2+^/A/G to the enzyme immobilization kinetics. To study the sole contribution of cobalt chelates, we blocked the support with glycine to remove the contribution of the aldehyde groups and performed the immobilization in the presence of high salt concentration to avoid ionic interactions (sample coordination chemistry; pink line in Figure 1). For the sole contribution of amine groups, we also blocked the aldehydes with glycine and performed the immobilization in the presence of imidazole (sample ionic chemistry; orange line in Figure 1). Finally, to study the sole contribution of the aldehydes, the immobilization was performed in the presence of a high concentration of salt and imidazole (sample covalent chemistry; purple line in Figure 1). This triheterofunctional support was designed to perform the enzyme immobilization in two sequential steps comprising a fast and first step of enzyme adsorption mainly driven by the cobalt chelates or by the secondary amine groups, which can help that the second immobilization step takes place (the enzyme covalent bonding mediated by glutaraldehyde moieties) by the gained proximity of the enzyme on the support’s surface. Figure 1a shows that the immobilization of His-BsADH is driven by the cobalt chelate groups as the immobilization rate was significantly reduced only when coordination chemistry was blocked by incubation with imidazole (purple line). For this enzyme, the aldehydes thus contribute to the first step of the immobilization to a lower extent than metal chelates. In the case of untagged TtNOX, we observe that cobalt chelates and aldehyde groups dominate the immobilization kinetics as the enzyme is immobilized at a similar rate regardless of whether interactions with one or the other group are blocked (Figure 1b). This insight agrees with the fact that aldehyde chemistry based on agarose activated with glyoxyl groups enables an efficient immobilization of that enzyme. Likewise, it has been reported that untagged TtNOX interacts nonspecifically with metal chelates through some exposed histidine residues at its surface.^34^ Finally, the immobilization of untagged BlCAT on AG-Co^2+^/A/G is dominated by the aldehyde and amine groups since the immobilization negligibly occurs when the aldehydes were blocked and the immobilization was performed in the presence of salt (Figure 1c). Blocking the interactions with the aldehydes slows down the immobilization to a similar extent as avoiding the electrostatic interactions between BlCAT and the amine groups of the support. Therefore, for the three enzymes herein studied and under this experimental setup, AG-Co^2+^/A/G drives the immobilization through a mixed mechanism. The type of interactions that dominate the immobilization kinetics of each enzyme therefore depends on their intrinsic physicochemical properties. Although the specific blocking of the different reactive groups allows us to understand the mechanisms that drive the immobilization of these three model enzymes, the fastest immobilization rates were achieved when the three reactive groups of AG-Co^2+^/A/G were available for the enzyme attachment.

In light of these results, the aldehyde groups do not play a fundamental role in the immobilization of BsADH in the first immobilization step; however, they may participate in a slower second step where a multivalent and irreversible attachment between the enzyme and the support is promoted. The irreversibility of this immobilization was confirmed by sodium dodecyl sulfate-polyacrylamide gel electrophoresis (SDS-PAGE) (Figure S4). Enzymes are undetectably lixiviated after incubating the immobilized preparations with 0.3 M imidazole, 1 M NaCl, or a combination of both. Under these conditions, the enzymes only bound through cobalt chelates and/or ionic interactions should be eluted to the bulk. However, partial enzyme lixiviation is only observed when incubating the immobilized biocatalysts at denaturing conditions (10 min boiling in β-mercaptoethanol-SDS Laemmli’s lysis buffer). This fact may be related to the subunit leaching of the oligomeric enzymes due to a suboptimal geometric congruence with the support surface (Figure S4).

### Spatial Distribution of Immobilized Enzymes across Differently Activated Supports

**Figure 2:**
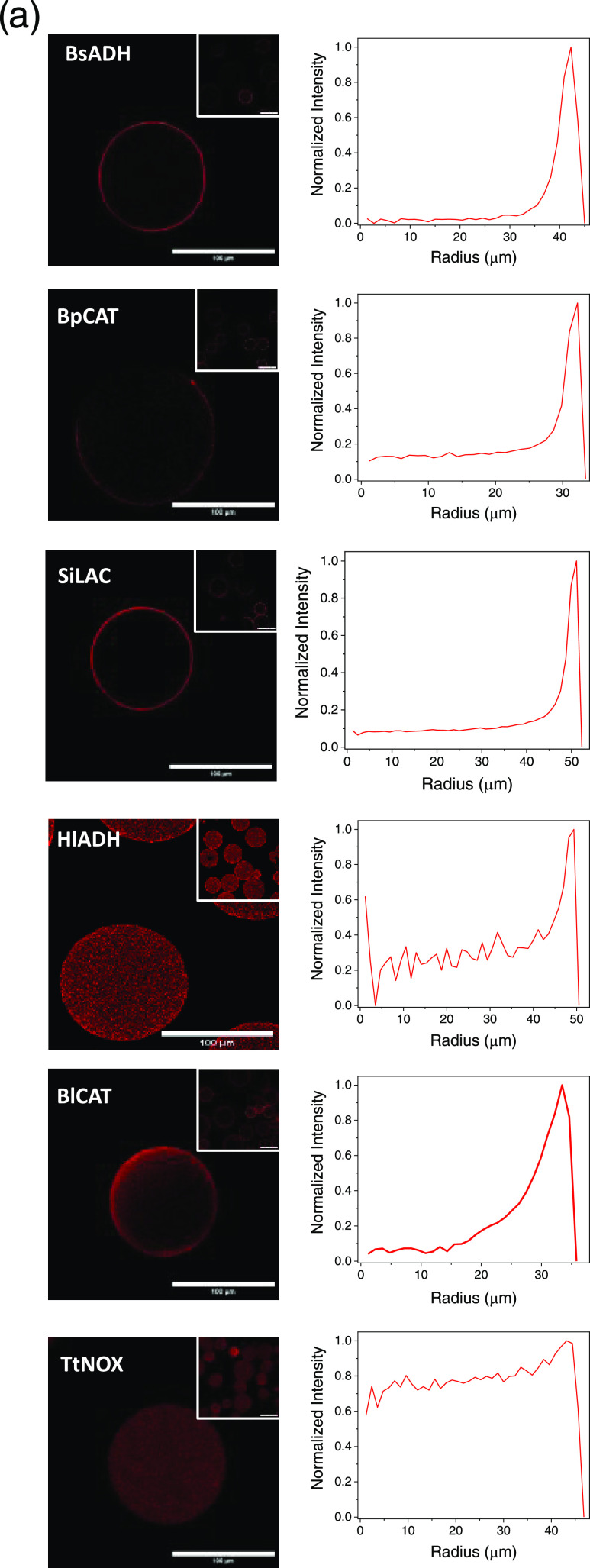

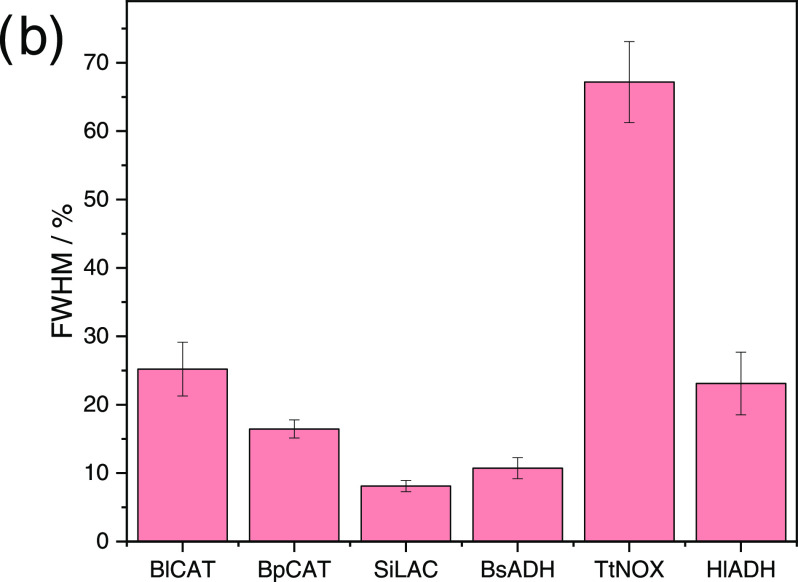
(a) Confocal fluorescence microscopy presenting the spatial organization of different enzymes labeled with rhodamine B (red channel, λ_ex_: 561 nm) inside AG-Co^2+^/A/G and the corresponding radial profiles. BsADH, BpCAT, and SiLAC are His-tagged at their N-terminus, while HlADH, BlCAT, and TtNOX are untagged. (b) Infiltration penetration percentage of each immobilized enzyme across the surface of different porous supports (see Experimental Methods).

To study the spatial distribution of different enzymes (BlCAT, BpCAT, BsADH, HlADH, TtNOX, and SiLAC) across the inner surface of the trifunctional support, we labeled the enzymes with a fluorescent probe (rhodamine isothiocyanate) prior to their immobilization. Then, we immobilized the labeled enzymes on AG-Co^2+^/A/G to investigate the enzyme distribution along the microparticle by confocal laser scanning microscopy (CLSM). From the CLSM images, we calculated the relative infiltration distance defined as the percentage of radius where more than 50% of maximum fluorescence intensity of the sample is detected according to Diamanti et al.^28^ To note, beads without enzymes showed no intrinsic fluorescence under the same conditions, discarding any artifact in the interpretation of the results (Figure S5). His-tagged enzymes (BpCAT, BsADH, and SiLAC) are located at the very outer surface of the porous agarose microbeads colonizing less than 15% of the particle radius (Figure 2). On the contrary, the untagged enzymes colonize inner regions of the beads, occupying up to 67% of the bead radius in the case of TtNOX. The spatial organization found for the different enzymes is supported by their immobilization kinetics. As previously reported by our group,^43^ high immobilization rates lead the enzymes to colonize the outer surface of porous materials since the enzyme immobilization is faster than the protein diffusion throughout the beads. On the contrary, low immobilization rates promote enzyme infiltration toward the inner regions of the beads by the protein diffusion is equal to or faster than the immobilization process. According to this, a His-tagged enzyme like BsADH only colonizes the most outer 5 μm of the bead radius (10% relative infiltration distance) thanks to its fast immobilization (100% yield in 5 min; Figure 1a). In contrast, the untagged enzymes colonize more inner regions due to their lower immobilization kinetics. For example, TtNOX is uniformly distributed across the radius of the beads because its immobilization rate is rather low (80% yield in 60 min; Figure 1b). This spatial organization of TtNOX explains its low recovered activity upon immobilization on this trifunctional support as oxygen mass transport restrictions are more severe when the enzyme is located at the inner regions of porous supports. A similar insight was observed when TtNOX was uniformly distributed across aldehyde-activated agarose microbeads.^39^ Regarding other untagged enzymes, we find the same trend for BlCAT (Figure 1c), yet this enzyme is infiltrated to a lower extent than TtNOX (50 kDa), likely due to its higher molecular weight (240 kDa) that may hamper its diffusion across the porous structure of the support.

### Stability and Structural Analysis of Immobilized Enzymes on Triheterofunctional Supports

To better explain the effect of the immobilization on the enzyme properties, we used a set of biophysical techniques to elucidate the structural rearrangements undergone in six different enzymes (BsADH, HlADH, TtNOX, BlCAT, BpCAT, and SiLAC) when immobilized on AG-Co^2+^/A/G. On one hand, we studied the intrinsic fluorescence spectrum of both immobilized and free enzymes (Figure S6) to acquire information about their microenvironment within the protein structure.^44^ On the other hand, we determined the relative anisotropy of free and immobilized enzymes labeled with fluorescein B isocyanate. The fluorescent anisotropy of small fluorophores tethered to the enzyme structure informs us about the apparent mobility of the protein through its rotational tumbling. The relative anisotropy of immobilized enzymes with respect to the anisotropy of their free counterpart reflects the changes in protein mobility promoted by the immobilization process. Normally, this relative anisotropy is greater as more stable the immobilized enzyme is, thus presenting a positive correlation with the half-life time of the immobilized biocatalysts.^45^ Finally, we determined the unfolding transition temperature (*T*_m_) of both free and immobilized enzymes by a thermal shift assay. All of these data, together with the half-life time of the free and immobilized enzymes, are compiled in Table 2 for comparative purposes.

**Table 2:** Stabilization of Immobilized Enzymes on AG Heterofunctional Activated Agarose Microbeads

enzyme	immobilization support	Δλ_max_ (nm)	*T*_m_ (°C)	half-life time (h)^a^	anisotropy
BsADH	free	330	73	3.8	1
	AG-Co^2+^/A/G	0	70	0.51	2.35
HlADH	free	335	51	2.5	1
	AG-Co^2+^/A/G	0	58	54	1.14
TtNOX	free	335	78	3.6	1
	AG-Co^2+^/A/G	30	84	9.2	4.62
BlCAT	free	330	49	4.2	1
	AG-Co^2+^/A/G	0	57	4.4	2.18
BpCAT	free	330	56	14.5	1
	AG-Co^2+^/A/G	–30	61	17	1.61
SiLAC	free	335	53	3.7	1
	AG-Co^2+^/A/G	0	62	6.7	2.23

a Half-life time studies were assayed at different temperatures accordingly with each enzyme; thus, 65 °C for BsADH, 45 °C for HLAHD, 80 °C for TtNOX, 40 °C for BpCAT, 45 °C for BlCAT, and 50 °C for SiLAC. BsADH, BpCAT, and SiLAC, are His-tagged at their N-terminus, while HlADH, BlCAT, and TtNOX are untagged.

The immobilized preparation of the two ADHs, BlCAT and SiLAC, presents the same λ_max_ values as their free counterparts, indicating that the enzyme structure suffers negligible changes upon the immobilization process. In contrast, all of these immobilized enzymes experience a reduction in their protein mobility (rotational tumbling) and enhancement of their *T*_m_ values and half-lives, except BsADH, which is less stable than the free enzyme although the anisotropy of the immobilized one was doubled. Specifically, the immobilized TtNOX presented a red-shifted λ_max_, which suggests that their aromatic residues are more exposed to the solvent upon the immobilization process. The more solvent-accessible conformation of the immobilized TtNOX exhibits a *T*_m_ 6 °C higher than the soluble form. This conformational change is accompanied by a reduced enzyme rotational tumbling supported by an anisotropy value almost 5 times higher than its free counterpart. Oppositely, the immobilization of BpCAT on AG-Co^2+^/A/G results in a blue-shifted λ_max_ in comparison with its free counterpart, suggesting that its interactions with the support promote less solvent-exposed aromatic residues. In this case, this interaction seems to be beneficial for BpCAT folding stability as the *T*_m_ of the immobilized enzyme is 5 °C higher than the free one. Moreover, the higher *T*_m_ values align with the higher half-life times under thermal inactivation. In summary, almost all tested enzymes are stabilized upon their immobilization on AG-Co^2+^/A/G as reflected in their increased *T*_m_ values and half-life times, except BsADH, which shows lower thermal and folding stabilities than its free counterpart.

All assembled biocatalysts show higher anisotropy values than the free enzymes, indicating that the immobilization decreases the enzyme local mobility (rotational tumbling). Indeed, we find a trend between the anisotropy values and the thermodynamic and kinetic stability of the immobilized enzymes. *T*_m_ and half-life times increase when the relative anisotropy does except for the BsADH biocatalysts. In most enzymes, the stabilization effects were accompanied by a reduction of the enzyme mobility within the porous microenvironment provided by the immobilization process and reflected in their augmented anisotropy values. In contrast, BsADH is more unstable when its rotational mobility is limited, suggesting that less flexible conformation fixed upon its immobilization on AG-Co^2+^/A/G is less thermally stable.

### Coimmobilization of Multienzyme Systems

Once we characterized the separately immobilized enzymes, we assembled the multienzyme system formed by BsADH, TtNOX, and BlCAT coimmobilizing them on the same AG-Co^2+^/A/G microparticle. Initially, we evaluated the effect of the enzyme immobilization order on the biocatalyst activity performance. To that aim, we prepared a sequentially coimmobilized heterogeneous biocatalyst (HB1) by first immobilizing TtNOX, followed by the BlCAT and lastly attaching the BsADH. Likewise, we prepared a coimmobilized heterogeneous biocatalyst with the three enzymes coimmobilized at the same time (HB2) (Table 3). The immobilization yield of TtNOX was lower when all three enzymes were coimmobilized simultaneously than when they were immobilized sequentially. This effect could be related to protein steric hindrances triggered by the fastest BsADH immobilization first colonizing the available matrix surface, thus exhibiting the same immobilization yields independently of the immobilization order (Table 3). The three enzymes recovered similar activities upon the immobilization regardless of whether they were immobilized sequentially (HB1) or simultaneously (HB2).

**Table 3:** Effect of the Immobilization Order on the Immobilization Parameters of Multienzyme Systems Coimmobilized on AG-Co^2+^/A/G

biocatalyst	immobilization order	enzymes	enzyme load (mg·g^–1^)	Ψ (%)^a^	recovered activity (U·g^–1^)^b^/(%)^c^
HB1	first	TtNOX	0.78	75	1.17 (14)
	second	BlCAT	0.78^d^	55	598 (11)
	third	BsADH	0.36	100	1.25 (42)
HB2	at the same time	TtNOX	0.53	51	0.93 (16)
		BlCAT	0.80^d^	57	696 (11)
		BsADH	0.36	100	1.23 (41)

a Immobilization yield, Ψ = (immobilized activity/offered activity) × 100.

b Recovered activity of the immobilized enzyme per gram of support after the immobilization process.

c (%) is defined as the coefficient between the specific activity of the immobilized enzymes and the specific activity of the soluble ones.

d Total protein content. BsADH, is His-tagged at its N-terminus, while BlCAT and TtNOX are untagged.

**Figure 3:**
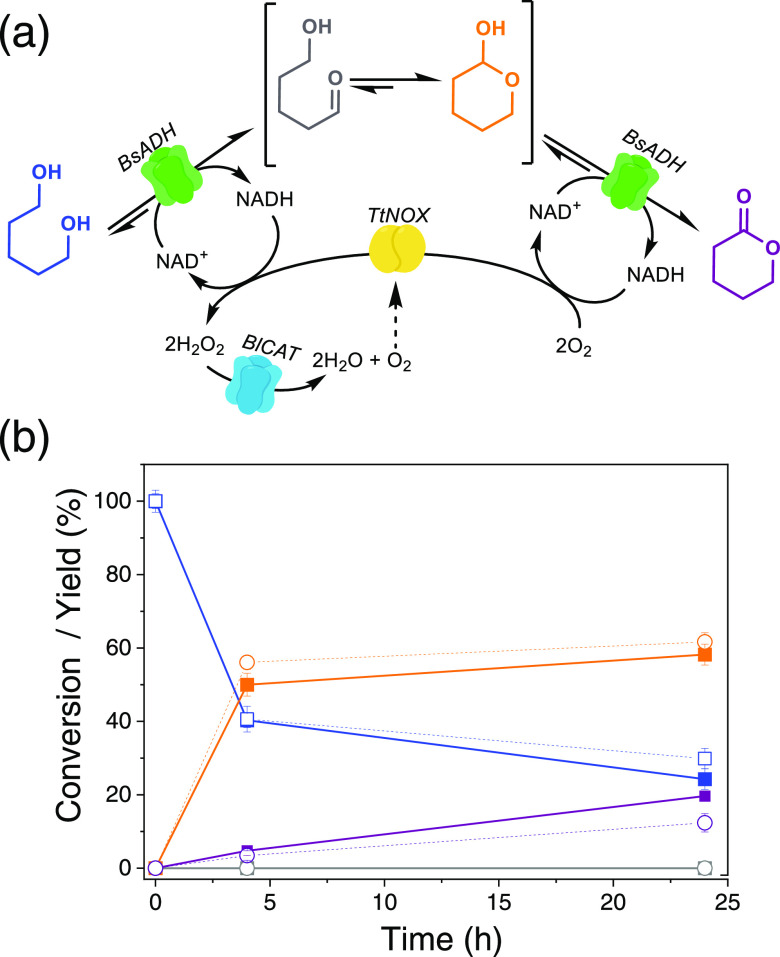
(a) Reaction scheme of the selective oxidation of 1,5-pentanediol integrating NAD^+^ recycling and H_2_O_2_ removal systems. (b) Time courses of the 1,5-pentanediol oxidation catalyzed by trienzyme systems coimmobilized on AG-Co^2+^/A/G either sequentially (solid lines and full squares) or simultaneously (dashed lines and empty circles). 1,5-Pentanediol (blue line), 5-hydroxypentanal (gray line), tetrahydro-2*H*-pyran-2-ol (orange line), and δ-valerolactone (magenta line). In all cases, reactions were performed by incubating 50 mg of heterogeneous biocatalyst with 300 μL of reaction mixture composed by 20 mM 1,5-pentanediol, 1 mM NAD^+^, 0.15 mM FAD^+^ in 100 mM sodium phosphate buffer pH 8 at 30 °C. BsADH, BpCAT, and SiLAC, are His-tagged at their N-terminus, while HlADH, BlCAT, and TtNOX are untagged.

Afterward, we evaluated both biocatalysts HB1 and HB2 under operational conditions by performing a model biotransformation. For that purpose, we applied HB1 and HB2 to selectively oxidize 1,5-pentanediol to its corresponding products (5-hydroxypentanal, tetrahydro-2*H*-pyran-2-ol, and δ-valerolactone) in batch operation conditions according to the enzyme selectivity previously reported by our group.^19^ We selected this model biocascade since it allows us to evaluate the coupling efficiency of the three enzymes to simultaneously oxidize the substrate, recycling a cofactor; the NAD^+^, and removing a toxic byproduct; the H_2_O_2_ (Figure 3a). After 24 h, both heterogeneous biocatalysts consumed more than 70% of the initial substrate 1,5-pentanediol, yielding a similar product profile, where tetrahydro-2*H*-pyran-2-ol was the major product (around 60%) (Figure 3b). In agreement with the recovered activity (Table 3), the performance of the multienzyme system was negligibly affected by the coimmobilization order. However, after 5 h, the reactions reach a plateau for the consumption of the diol, suggesting the partial inactivation of the BsADH.

A spectrophotometric assay confirmed that BsADH is dramatically inactivated upon 8 h of operational use, maintaining only 20% of its initial activity after 24 h (Figure S7). Despite this inactivation issue, the coimmobilized multienzyme system (HB2) reaches an 18% higher substrate consumption after 24 h than their soluble and separately immobilized counterpart enzymes, supporting the fact that the catalytic efficiency increases when the multienzyme system is immobilized within the same confined space (Figure S8).

### Expanding the Functionalization Chemistry to Other Materials

Apart from agarose microbeads, we expanded the developed functionalization chemistry to other typically employed materials for enzyme immobilization. To this aim, we functionalized commercially available methacrylate microbeads and macroporous cellulose beads with the same active groups than AG-Co^2+^/A/G (referred to as Pu-Co^2+^/A/G and CE-Co^2+^/A/G, respectively). Then, we simultaneously coimmobilized BsADH, TtNOX, and BlCAT on these two other materials to evaluate their performance (Table 4). BsADH achieves similar immobilization yield on AG-Co^2+^/A/G and Pu-Co^2+^/A/G but slightly lower on CE-Co^2+^/A/G. Moreover, the cellulose-based carrier promoted a dramatic inactivation of this enzyme upon the immobilization. TtNOX behaves very similar when coimmobilized on hydrophilic matrices as AG-Co^2+^/A/G and CE-Co^2+^/A/G but recovering 1.8 times higher activity when immobilized on a cellulose matrix. In contrast, this enzyme achieves higher immobilization yields when immobilized on Pu-Co^2+^/A/G but recovers 2.3 times lower activity upon its immobilization on this hydrophobic support. According to our previous results,^39^ the hydrophobicity of the support surfaces favors the immobilization of TtNOX at the expense of enzyme inactivation. Finally, BlCAT attains different immobilization yields depending on the matrix composition but expresses two times higher specific activity on agarose-based supports than on cellulose and methacrylate ones. In summary, all enzymes recovered the highest activities upon the immobilization on the most hydrophilic support (agarose microbeads) herein tested. Therefore, the physicochemical properties of the support directly affect the performance of the immobilized enzymes, even though they are immobilized through the same chemistry.

**Table 4:** Coimmobilization of Multienzyme Systems on Triheterofunctional Supports

biocatalyst	support	enzymes	enzyme load (mg·g^–1^)	Ψ (%)^a^	recovered activity (U·g^–1^)/(%)^b^
HB2-AG	AG-Co^2+^/A/G	TtNOX	0.53	51	0.93 (16)
		BlCAT	0.80^c^	57	626 (11)
		BsADH	0.36	100	1.23 (41)
HB2-Pu	Pu-Co^2+^/A/G	TtNOX	0.68	97	0.95 (13)
		BlCAT	0.75^c^	74	254 (5)
		BsADH	0.30	100	0.84 (34)
HB2-CE	CE-Co^2+^/A/G	TtNOX	0.35	50	1.1 (29)
		BlCAT	0.36^c^	35	107 (4)
		BsADH	0.30	73	0.068 (3)

a Immobilization yield, Ψ = (immobilized activity/offered activity) × 100.

b Recovered activity of the immobilized enzyme (%) is defined as the coefficient between the specific activity of the immobilized enzymes and the specific activity of the soluble ones.

c Total protein content. BsADH is His-tagged at its N-terminus, while BlCAT and TtNOX are untagged.

### Biocatalyst Recycling

**Figure 4:**
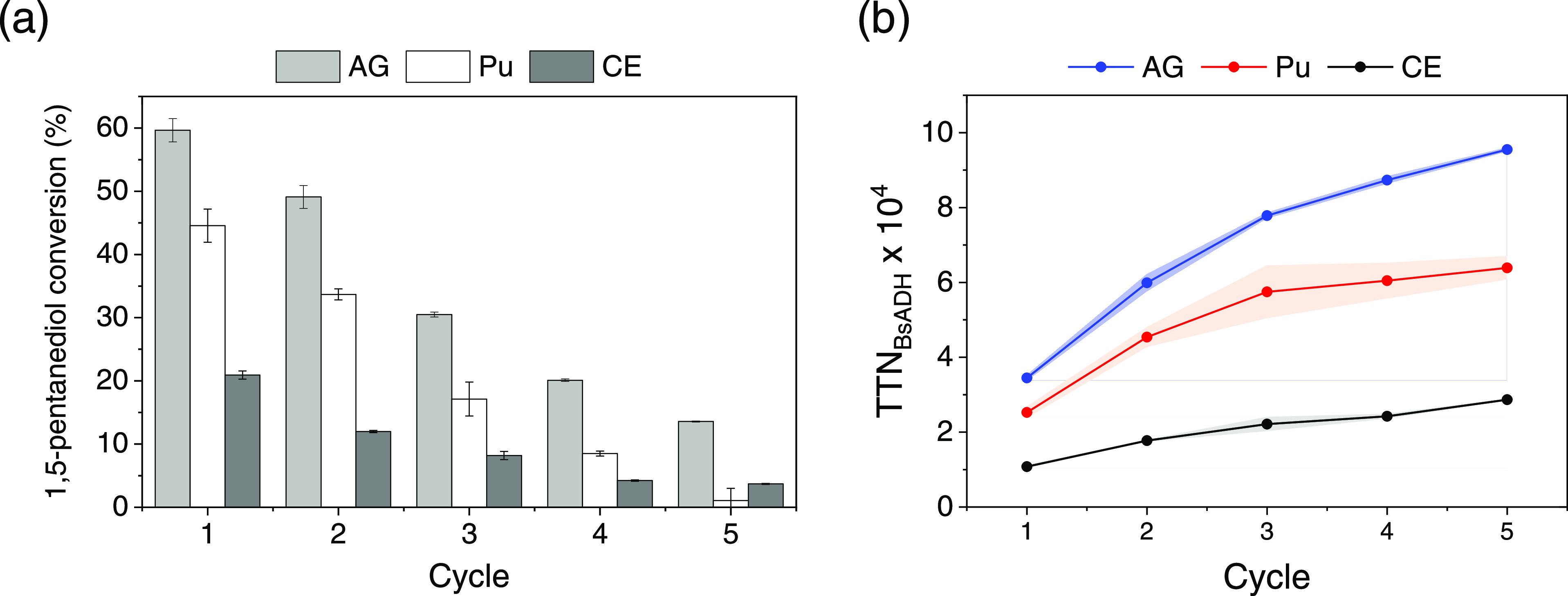
Recycling of coimmobilized heterogeneous biocatalysts during the oxidation of 1,5-pentanediol. (a) Each cycle corresponds to 24 h working at 20 mM 1,5-pentanediol, 1 mM NAD^+^, and 0.15 mM FAD^+^ in 100 mM sodium phosphate buffer pH 8 at 30 °C. (b) Accumulated TTN of BsADH during recycling, defined as the mol of oxidized 1,5-pentanediol and tetrahydro-2*H*-pyran-2-ol per mol of tetrameric BsADH after the fifth cycle; standard deviation is depicted in the shadows of the same color.

As the last part of our study, we compared the reusability performance of HB2-AG, HB2-Pu, and HB2-CE during the oxidation of the same model substrate in repeated batch cycles (Figure 4). The agarose-based biocatalyst (HB2-AG) shows a higher yield and operational stability among the supports studied. The first cycle conversion of 1,5-pentanediol was 1.3 and 2.8 times larger when the multienzyme system is immobilized on AG-Co^2+^/A/G than on Pu-Co^2+^/A/G and CE-Co^2+^/A/G, respectively. Remarkably, the system immobilized on agarose microbeads maintains more than 80% of its initial activity after the second batch reaction cycle. Hence, this multifunctional heterogeneous biocatalyst is stable for more than 48 h of discontinuous operation as each reaction cycle corresponds to 24 h of reaction at pH 8 and 30 °C. In the three supports herein analyzed, the decrease of the product yield along the cycles is supported by the dramatic inactivation found for the coimmobilized BsADH and BlCAT after the fifth batch cycle (Table S6). However, the further stabilization of these enzymes under these operational conditions is out of the scope of this work. Previously, BsADH was thermostabilized by its immobilization on agarose supports functionalized with epoxy and cobalt chelates; noteworthily, the BsADH thermostabilization did not afford enhanced operational stability.^19^ The highest operational stabilization achieved when the multienzyme system is immobilized on AG-Co^2+^/A/G leads to an accumulated total turnover number (TTN, defined as the mol of oxidized 1,5-pentanediol and tetrahydro-2*H*-pyran-2-ol per mol of tetrameric BsADH) of 1 × 10^5^ after five batch cycles (Figure 4b). This TTN is 333% higher than the same multienzyme system immobilized on CE-Co^2+^/A/G. Expectedly, the accumulated TTN reaches a plateau upon the fourth cycle due to the inactivation of the enzymes. To note, the lower turnover of the multienzyme system immobilized on the cellulose-based support is attributed to the low recovered activity of BsADH upon its immobilization on CE-Co^2+^/A/G (Table 4).

## Conclusions

We describe the preparation of a heterofunctional support that enables the coimmobilization of a variety of enzymes requiring different immobilization chemistries. The herein-characterized support possesses three chemical functionalities, namely, amino, aldehyde, and cobalt moieties, which synergistically permit a fast irreversible enzyme immobilization at neutral pH values of His-tagged and untagged enzymes. Moreover, it is also possible to change the chemical nature of the aldehyde moiety by replacing it with an epoxide or aromatic aldehyde such as hydroxymethylfurfural. However, we found that aldehyde groups as electrophiles to establish covalent bonds between the enzymes and the support outperform the other two (epoxides and HMF ones). With this information in hand, we exploited this trifunctional carrier to coimmobilize a trienzyme system for the regioselective oxidation of 1,5-pentanediol to its corresponding lactol and lactone derivatives. Additionally, we also showed the possibility to expand this surface chemistry to different porous materials such as cellulose and methacrylate microbeads; however, the physicochemical properties of the support surface impact the operational performance and stability of the coimmobilized systems. Thus, this trifunctional support demonstrates its versatility to coimmobilize a wide variety of different enzymes under mild immobilization conditions, opening the possibility to coimmobilize multienzyme systems aimed at enhancing the efficiency of cascade biotransformations.

## Supporting Information Available

Supporting schemes including the preparation of AG-Co^2+^/A/G, AG-Co^2+^/A/E, and AG-Co^2+^/H; supporting figures including the distribution of agarose-activated microbeads with different functionalities, immobilization and thermal inactivation kinetics, enzyme desorption assays, confocal fluorescence microscopy images of AG-Co^2+^/A/G, spectra of intrinsic protein fluorescence, residual activity of HBs under operation conditions and consumed 1,5-pentanediol after 24 h by the soluble or coimmobilized enzymes; supporting tables including the activation degree of agarose microbeads, heterofunctional activation of agarose microbeads, abbreviation of the differently prepared supports, single-enzyme immobilization parameters on AG-Co^2+^/E microbeads, and thermal stability of soluble enzymes and individual residual activity of coimmobilized enzymes after five batch cycles (PDF)

## Abbreviations

ADH: alcohol dehydrogenase
BsADH: alcohol dehydrogenase from *Bacillus stearothermophilus*
HlADH: alcohol dehydrogenase from horse liver
TtNOX: NADH oxidase from *Thermus thermophilus*
BlCAT: catalase from bovine liver
BpCAT: catalase from *Bordetella pertussis*
SiLAC: lactonase from *Sulfolobus islandicus*
HB: heterogeneous biocatalyst
E: epoxy groups
EDA: ethylenediamine
IDA: iminodiacetic acid
GA: glutaraldehyde
BD: 1,4-butanediol diglycidyl ether
HMF: hydroxymethylfurfural
A: amine groups
G: aldehyde groups
H: hydrophobic aldehyde groups
AG-E: epoxy-activated AG
AG-IDA/E: AG functionalized with epoxide and IDA groups
AG-Co^2+^/A/G: AG functionalized with GA, EDA, and IDA/cobalt groups
AG-Co^2+^/A/E: AG functionalized with BD epoxide, EDA, and IDA/cobalt groups
AG-Co^2+^/H: AG functionalized with HMF and IDA/cobalt groups
AG: 4% cross-linked agarose beads
Pu: epoxy methacrylate microbeads ECR8204F
CE: cellulose MT200 microbeads
NAD^+^: nicotinamide adenine dinucleotide
NADH: reduced nicotinamide adenine dinucleotide
FAD^+^: flavin adenine dinucleotide
*K*_M_: Michaelis–Menten constant
TTN: total turnover number
