# Supplementary material for: Multienzyme Coimmobilization on Triheterofunctional Supports
Source: Biomacromolecules. 2023 Jan 17;24(2):929–42. doi: 10.1021/acs.biomac.2c01364 (PMC10018741; doi:10.1021/acs.biomac.2c01364)
Supplement: Supplementary file 1 — bm2c01364_si_001.pdf [file bm2c01364_si_001.pdf]

Supporting Information

Multienzyme coimmobilization on triheterofunctional supports

Javier Santiago-Arcos[a]‡, *Susana Velasco-Lozano*[a,b,c]‡* *and Fernando López-Gallego*[a,d]*

[a] Heterogeneous Biocatalysis Laboratory, CIC biomaGUNE, Edificio Empresarial "C", Paseo de Miramón 182, 20009 Donostia, Spain.
[b] Instituto de Síntesis Química y Catálisis Homogénea (ISQCH-CSIC), Universidad de Zaragoza, C/ Pedro Cerbuna, 12, 50009, Zaragoza, Spain.
[c] Aragonese Foundation for Research and Development (ARAID), 50018 Zaragoza, Spain.
[d] IKERBASQUE, Basque Foundation for Science, 48009 Bilbao, Spain.
‡Equal contribution.

*Corresponding authors (F. López-Gallego) and (S. Velasco-Lozano)
Phone: +34 943003500 Ext 309, Fax: +34 943003501
Email: flopez@cicbiomagune.es and svelasco@unizar.es

Content

Scheme S1. Preparation pathway of AG-Co^{2+}/A/G. 3
Scheme S2. Preparation pathway of AG-Co^{2+}/A/E.. 3
Scheme S3. Preparation pathway of AG-Co^{2+}/H. 4
Figure S1. Distribution of agarose-activated microbeads with different functionalities 5
Figure S2. Enzyme immobilization kinetics on different triheterofunctional supports................ 6
Figure S3. Thermal inactivation kinetics 7
Figure S4. Enzyme desorption assays... 8
Figure S5. Confocal fluorescence microscopy images of AG-Co^{2+}/A/G 9
Figure S6. Spectra of intrinsic protein fluorescence before the immobilization 10
Figure S7. Residual activity of HB1 and HB2 under operation conditions 11
Figure S8. Consumed 1,5-pendanediol after 24 h. 11
Table S1. Activation degree of agarose-microbeads.. 12
Table S2. Heterofunctional activation of agarose microbeads 12
Table S3. Abbreviation of the differently prepared supports 13
Table S4. Single-enzyme immobilization parameters on AG-Co^{2+}/E microbeads. 13
Table S5. Thermal stability of soluble enzymes. 13
Table S6. Individual residual activity of coimmobilized enzymes after five batch cycles. 14
References …………………………………………………………………………..……..14

Supporting Schemes

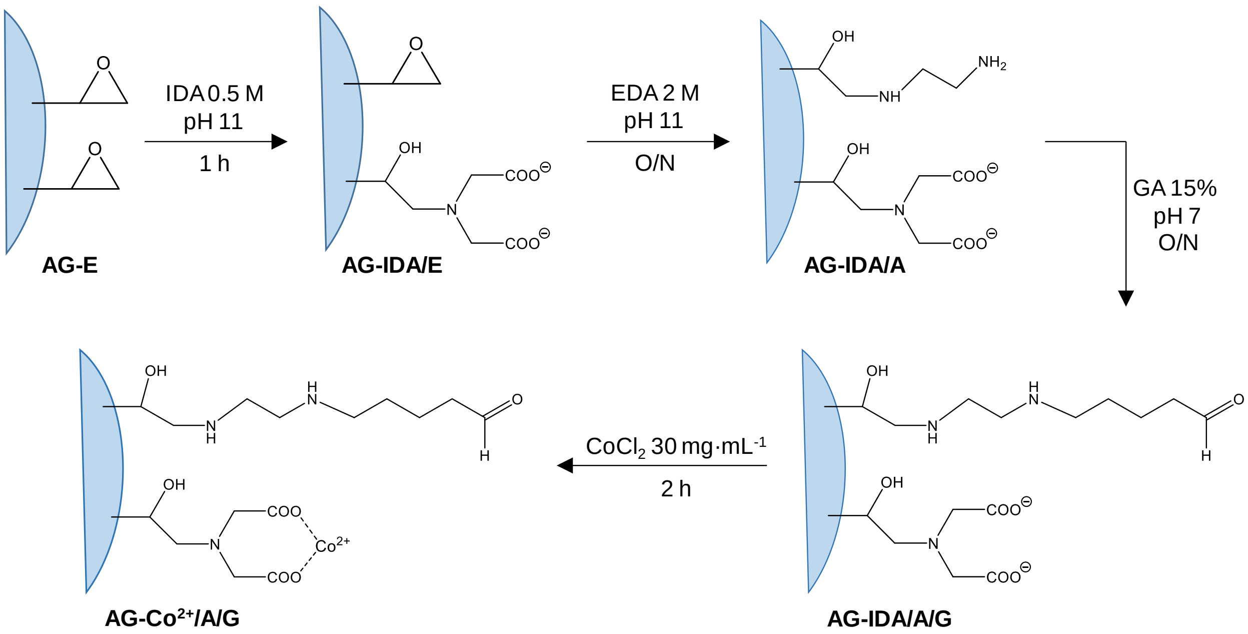

Scheme S1. Preparation pathway of AG-Co^{2+}/A/G.

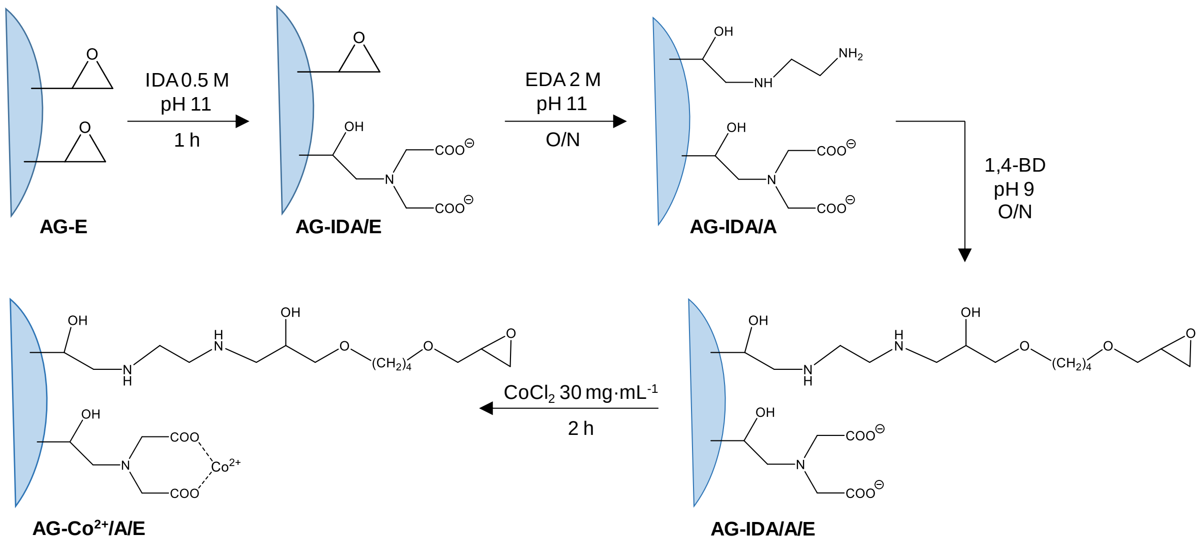

Scheme S2. Preparation pathway of AG-Co^{2+}/A/E.

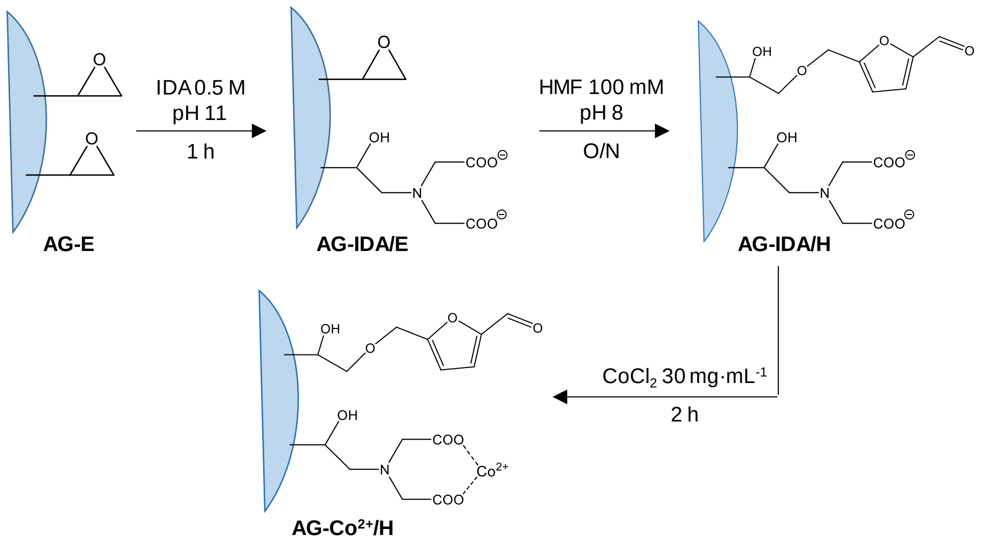

Scheme S3. Preparation pathway of AG-Co^{2+}/H.

Supporting Figures

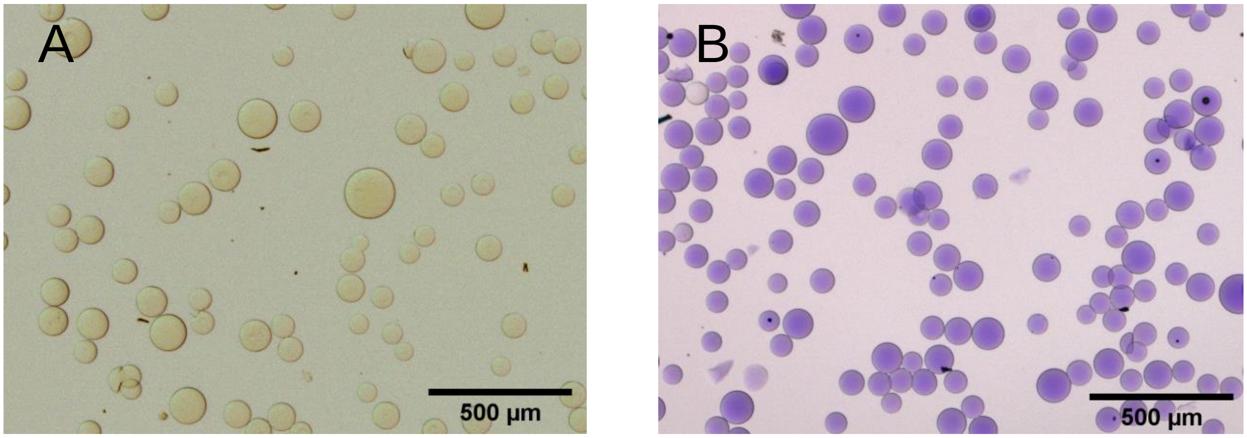

Figure S1. Distribution of agarose-activated microbeads with different functionalities. A) AG-A/IDA. B) AG-A/G/IDA.

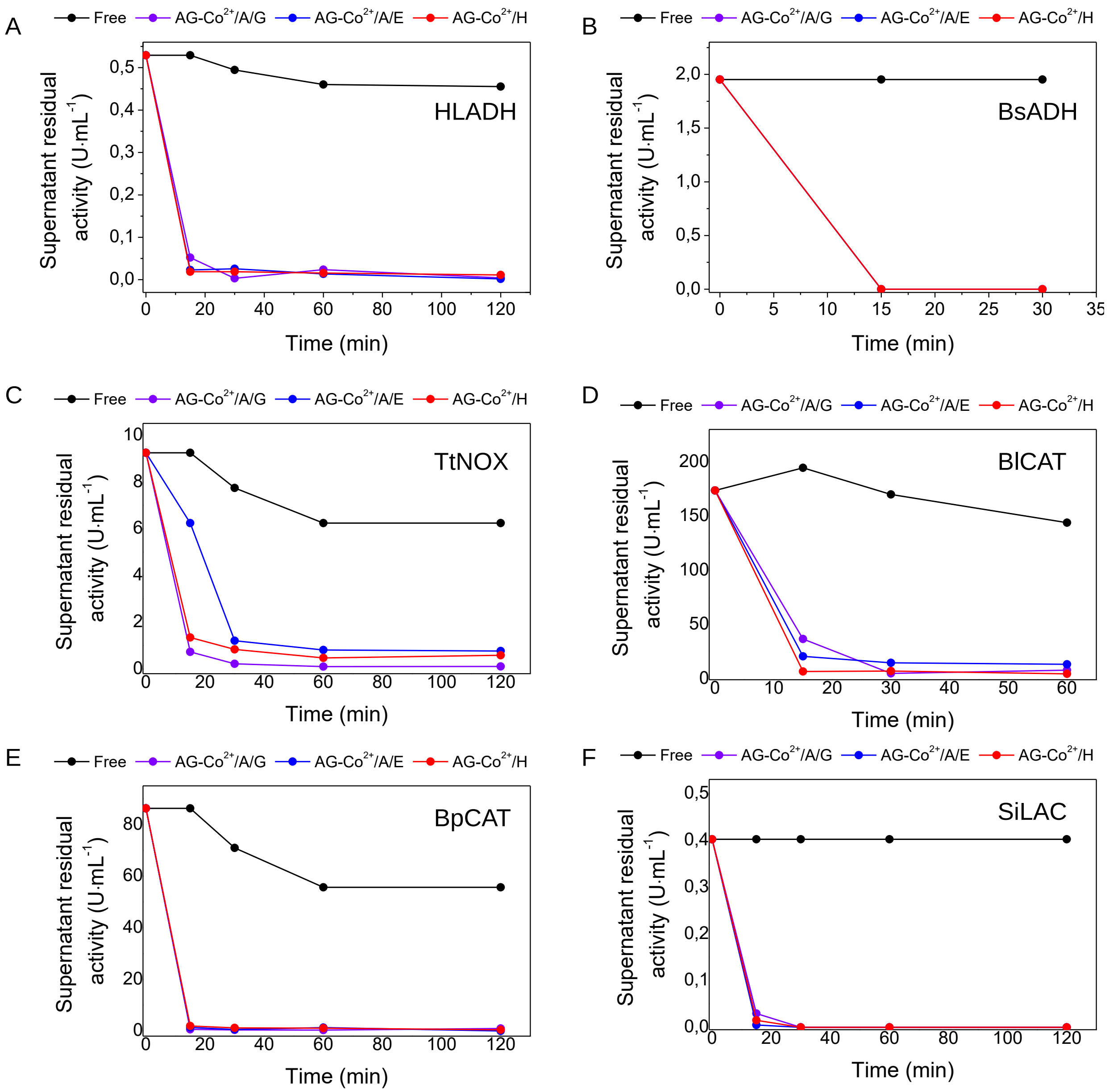

Figure S2. Enzyme immobilization kinetics on different triheterofunctional supports. The immobilization was conducted by mixing 10 mL of enzyme solution (in 100 mM sodium phosphate buffer pH 7) with 1 g of support (AG-Co^{2+}/A/G or AG- Co^{2+}/A/E or AG-Co^{2+}/H) and maintained under gentle agitation at 25 rpm at 4 °C. BsADH, BpCAT, and SiLAC, are His-tagged at their N-terminus, while HlADH, BlCAT, and TtNOX are untagged.

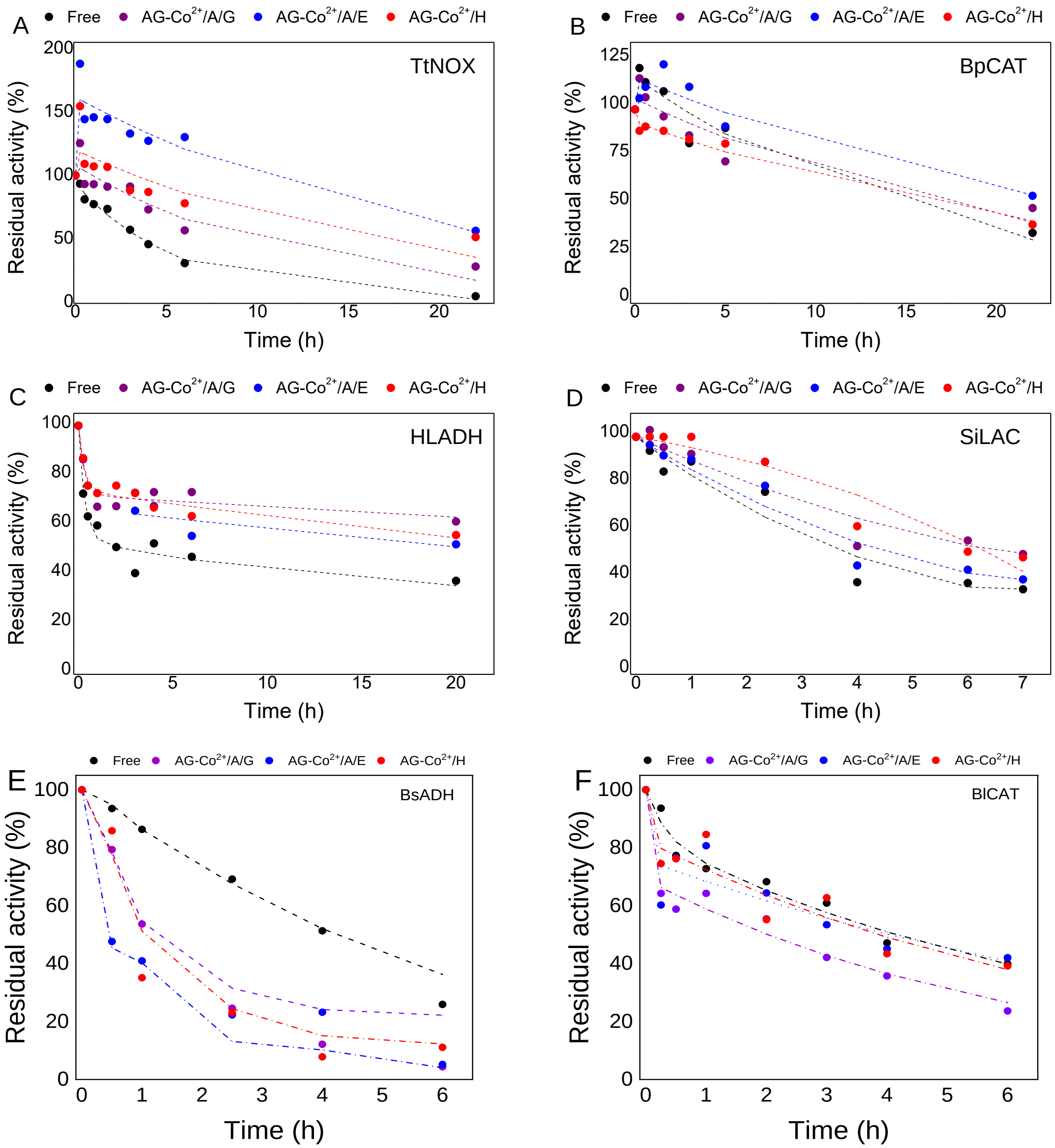

Figure S3. Thermal inactivation kinetics of soluble and immobilized enzymes on different heteroactivated supports. A) TtNOX at 80 ºC. B) BpCAT 40 ºC. C) HlADH at 45 ºC. D) SiLAC at 50 ºC E) BsADH at 70°C. F) BlCAT at 45°C. All thermal inactivations were conducted in 100 mM sodium phosphate buffer pH 8. Filled circles represent the obtained experimental measurements and the continuous dashed lines correspond to the fitting 3-parameters biexponential kinetic inactivation model. [1] BsADH, BpCAT, and SiLAC, are His-tagged at their N-terminus, while HlADH, BlCAT, and TtNOX are untagged.

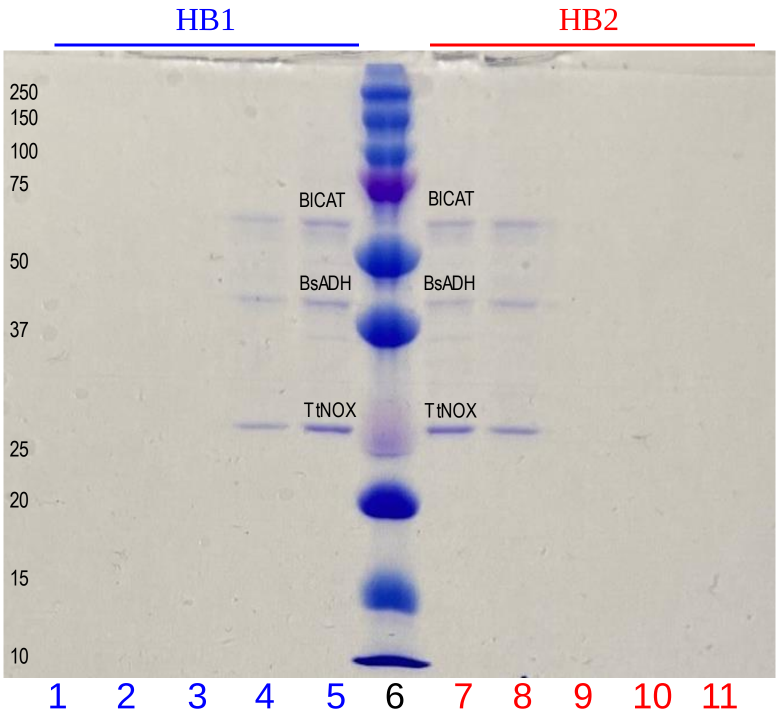

Figure S4. Enzyme desorption assays. Biocatalysts HB1 (sequentially coimmobilized enzymes) and HB2 (coimmobilized enzymes at the same time addition order) were incubated 1 h with 10 volumes of: A) 0.3 M imidazole pH 7; or B) 1 M NaCl; or C) 0.3 M imidazole and 1 M NaCl. After incubation, the suspensions were filtered and the eluted enzymes in the supernatant were analyzed by SDS-PAGE gel. Lanes 1-5 correspond to HB1. Lanes 7 to 11 correspond to HB2. Lanes: 1, 11: eluted enzymes at C conditions; 2, 20: eluted enzymes at B conditions; 3, 9: eluted enzymes at A conditions; 4, 8: Boiled HBs (1:10 suspension in 100 mM sodium phosphate buffer pH 8) in Laemmli buffer without any desorption treatment; 5, 7: Soluble enzymes; 6: Molecular weight marker. BsADH is His-tagged at its N-terminus, while BlCAT, and TtNOX are untagged.

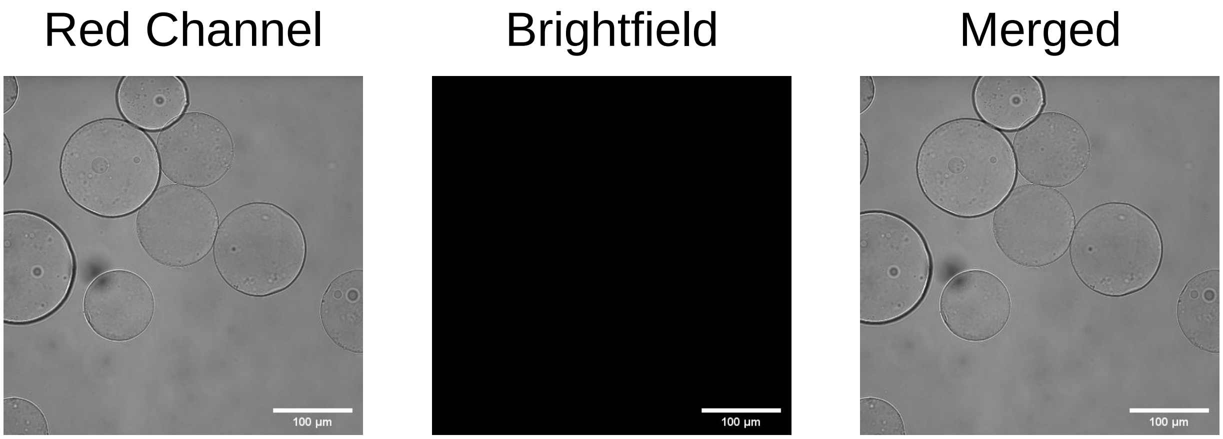

Figure S5. Confocal fluorescence microscopy images of AG-Co^{2+}/A/G (red channel, λ_{ex}: 561 nm; brightfield channel and merged channels). The material shows no intrinsic fluorescence under the same conditions the labeled enzymes do.

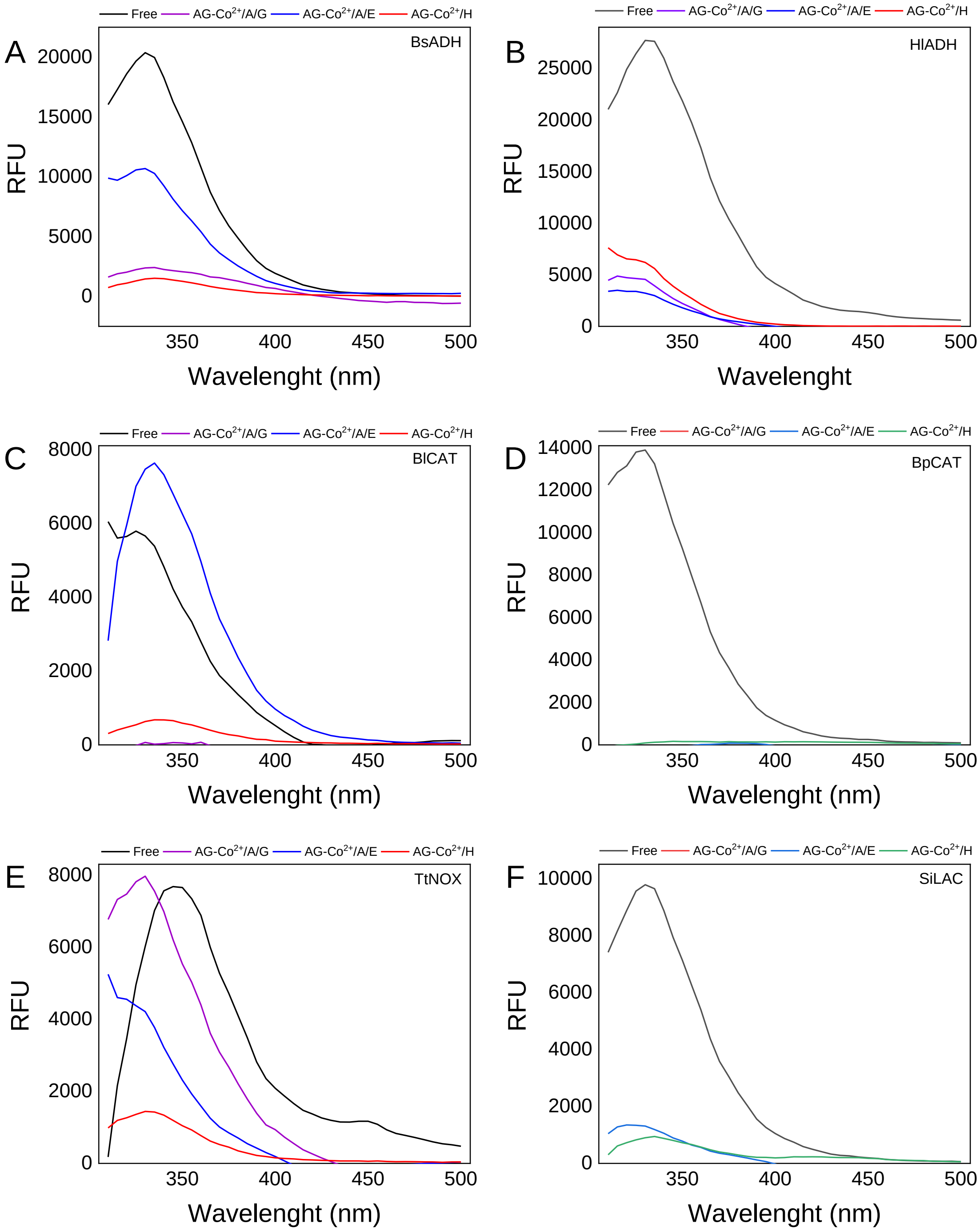

Figure S6. Spectra of intrinsic protein fluorescence before the immobilization (black lines, free) and upon the immobilization (purple, blue and red lines for AG-Co^{2+}/A/G, AG-Co^{2+}/A/E and AG-Co^{2+}/H, respectively). BsADH, BpCAT, and SiLAC, are His-tagged at their N-terminus, while HlADH, BlCAT, and TtNOX are untagged.

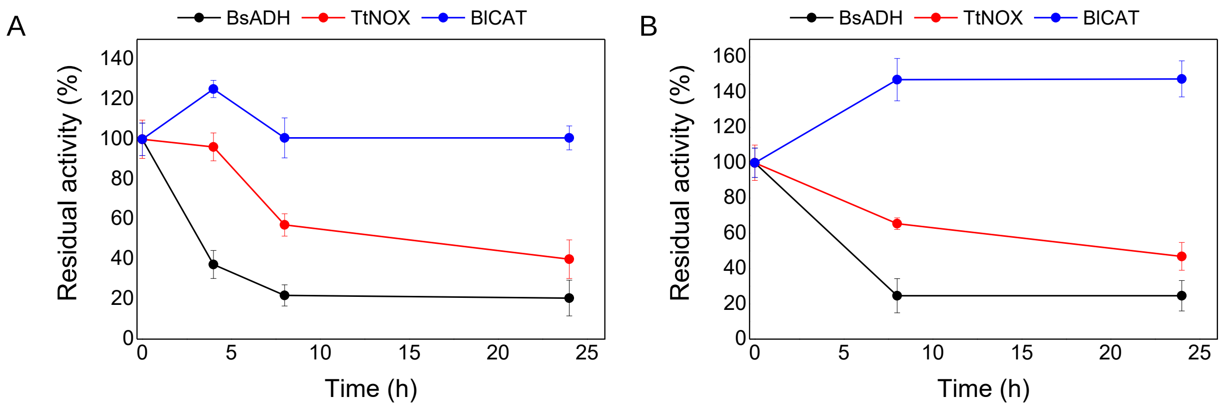

Figure S7. Residual activity of HB1 (A) and HB2 (B) under operation conditions. Reaction mixture consisted in 50 mg of HB and 0.3 mL of 20 mM 1,5-pentanediol, 1 mM NAD^+, 0.15 mM FAD^+ in 100 mM sodium phosphate buffer pH 8 at 30 ºC.

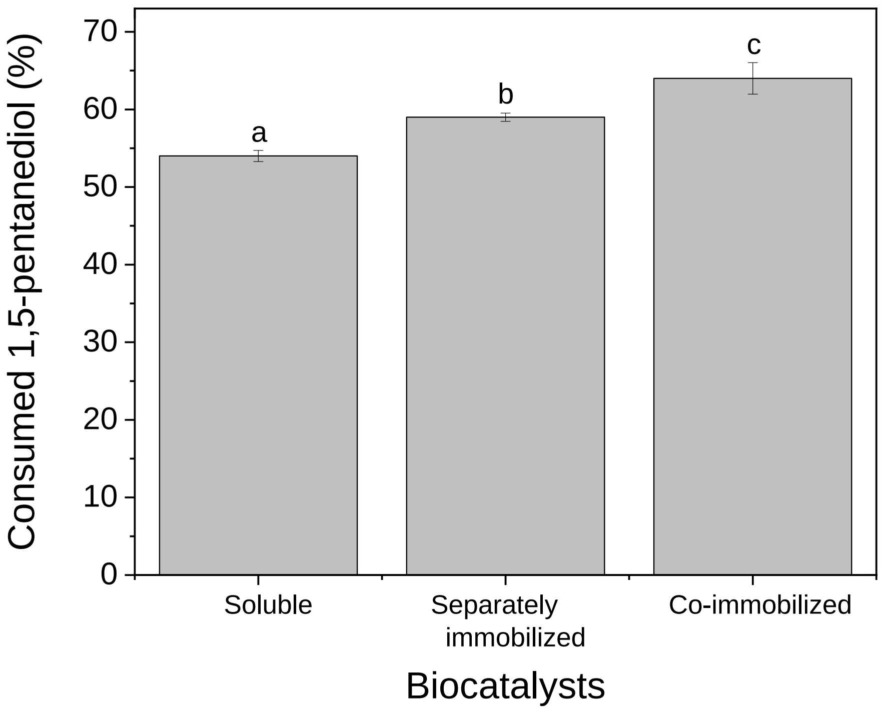

Figure S8. Consumed 1,5-pendanediol after 24 h. In both cases reactions were performed by incubating 50 mg of HB2 of its equivalent in soluble enzymes with 300 µL of reaction mixture composed by 20 mM 1,5-pentanediol, 1 mM NAD^+, 0.15 mM FAD^+ in 100 mM sodium phosphate buffer pH 8 at 30 ºC. [a,b,c] Means with different letter are significantly different ($P < 0.05$).

Supporting Tables

Table S1. Activation degree of agarose-microbeads 4 BCL

IDA (mM)	pH	incubation Time (h)	activation degree ($\mu mol \cdot g^{-1}$)	
			IDA	epoxy
500	11	0.5	22.2	16.5
500	9	1	5.2	33.5
500	11	1	20.2	18.5
500	9	3	25.3	13.4
500	11	3	26.0	12.7

Total epoxy-groups after epichlorhydrin treatment = 38.7 $\mu mol \cdot g^{-1}$
Total diols after epichlorhydrin treatment & H_2SO_4-hydrolysis = 82.2 $\mu mol \cdot g^{-1}$

Table S2. Heterofunctional activation of agarose microbeads

support	step	introduced functional group	activation degree ($\mu mol \cdot g^{-1}$)
AG-Co^{2+}/A/G	1	Epoxy	19 ± 1.5
	2	IDA	20 ± 2.1
	3	EDA	12 ± 2.6
	4	GA	26 ± 3.5
AG-Co^{2+}/A/E	1	Epoxy	18 ± 1.5
	2	IDA	20 ± 2.1
	3	EDA	13 ± 1.8
AG-Co^{2+}/H	1	Epoxy	18 ± 1.5
	2	IDA	20 ± 2.1
	3	HMF	4.3 ± 0.7

Table S3. Abbreviation of the differently prepared supports

abbreviation	functionalities	matrix	support description
AG-E	Monofunctional	Agarose	Epoxy activated agarose
AG-IDA	Monofunctional	Agarose	Iminodiacetic activated agarose
AG-IDA/E	Bifunctional	Agarose	Epoxy and iminodiacetic activated agarose
AG-IDA/A	Bifunctional	Agarose	Amino and iminodiacetic activated agarose
AG-IDA/A/G	Trifunctional	Agarose	Glutaraldehyde, amino and iminodiacetic activated agarose
AG-Co^{2+}/A/G	Trifunctional	Agarose	Glutaraldehyde, amino and cobalt activated agarose
AG-Co^{2+}/A/E	Trifunctional	Agarose	Epoxy, amino and cobalt activated agarose
AG-Co^{2+}/H	Bifunctional	Agarose	Hydroxymethylfurfural and cobalt activated agarose
Pu-Co^{2+}/A/G	Trifunctional	Methacrylate	Glutaraldehyde, amino and cobalt activated methacrylate
CE-Co^{2+}/A/G	Trifunctional	Cellulose	Glutaraldehyde, amino and cobalt activated cellulose

Table S4. Single-enzyme immobilization parameters on AG-Co^{2+}/E microbeads.

enzyme	Ψ (%) [a]	recovered activity (%) [b]
BsADH	100	23
HlADH	97	49
TtNOX	54	0
BlCAT	15	0
SiLAC	100	7.4

[a] Immobilization yield, Ψ = (immobilized activity/offered activity) x 100. [b] Recovered activity is defined as the coefficient between the specific activity of the immobilized enzymes and the specific activity of the soluble ones x 100. BsADH, BpCAT, and SiLAC, are His-tagged at their N-terminus, while HlADH, BlCAT, and TtNOX are untagged.

Table S5. Thermal stability of soluble enzymes.

soluble Enzyme	half-life time (h)	temperature (ºC) / Tm (ºC)
BsADH	3.8	65 /73
HlADH	2.5	45 / 51
TtNOX	3.6	80 / 78
BlCAT	4.2	45 / 49
BpCAT	14.5	50 / 56
SiLAC	3.9	50 / 53

In all cases enzymes were incubated at 100 mM sodium phosphate buffer pH 8 at the indicated temperature. Half-life times were obtained by fitting the experimental data to a 3-parameters biexponential kinetic inactivation model.[1] BsADH, BpCAT, and SiLAC, are His-tagged at their N-terminus, while HlADH, BlCAT, and TtNOX are untagged.

Table S6. Individual residual activity of coimmobilized enzymes after five batch cycles.

biocatalyst	support	enzyme	residual activity (%)
HB2-AG	AG-Co^{2+}/A/G		0
HB2-Pu	Pu-Co^{2+}/A/G	BsADH	7
HB2-CE	CE-Co^{2+}/A/G		1
HB2-AG	AG-Co^{2+}/A/G		24
HB2-Pu	Pu-Co^{2+}/A/G	TtNOX	27
HB2-CE	CE-Co^{2+}/A/G		27
HB2-AG	AG-Co^{2+}/A/G		9
HB2-Pu	Pu-Co^{2+}/A/G	BlCAT	6
HB-CE	CE-Co^{2+}/A/G		11

Each reaction cycle corresponds to 24 h working at 20 mM 1,5-pentanediol, 1 mM NAD^+, 0.15 mM FAD^+ in 100 mM sodium phosphate buffer pH 8 at 30 ºC. BsADH is His-tagged at its N-terminus, while BlCAT and TtNOX are untagged.

References

(1) Aymard, C.; Belarbi, A. Kinetics of thermal deactivation of enzymes: a simple three parameters phenomenological model can describe the decay of enzyme activity, irrespectively of the mechanism. *Enzyme Microb. Technol.* **2000**, *27* (8), 612-618.
